# Laminin as a Biomarker of Blood–Brain Barrier Disruption under Neuroinflammation: A Systematic Review

**DOI:** 10.3390/ijms23126788

**Published:** 2022-06-17

**Authors:** Juan F. Zapata-Acevedo, Valentina García-Pérez, Ricardo Cabezas-Pérez, Monica Losada-Barragán, Karina Vargas-Sánchez, Rodrigo E. González-Reyes

**Affiliations:** 1 Grupo de Investigación en Neurociencias (NeURos), Centro de Neurociencias Neurovitae-UR, Instituto de Medicina Traslacional (IMT), Escuela de Medicina y Ciencias de la Salud, Universidad del Rosario, Bogotá 111711, Colombia; juanfe.zapata@urosario.edu.co; 2 Grupo de Neurociencia traslacional, Facultad de Medicina, Universidad de los Andes, Bogotá 111711, Colombia; v.garciap@uniandes.edu.co (V.G.-P.); j.vargass@uniandes.edu.co (K.V.-S.); 3 Grupo de Investigación en ciencias Biomédicas GRINCIBIO, Facultad de Medicina, Universidad Antonio Nariño, Sede Bogotá D.C., Bogotá 110231, Colombia; ricardocabe@gmail.com; 4 Biología Celular y Funcional e Ingeniería de Moléculas, Departamento de Biología, Universidad Antonio Nariño, Sede Bogotá D.C., Bogotá 110231, Colombia; monica.losada@uan.edu.co

**Keywords:** blood–brain barrier, laminin, neuroinflammation, neurovascular unit, animal models, extracellular matrix

## Abstract

Laminin, a non-collagenous glycoprotein present in the brain extracellular matrix, helps to maintain blood–brain barrier (BBB) integrity and regulation. Neuroinflammation can compromise laminin structure and function, increasing BBB permeability. The aim of this paper is to determine if neuroinflammation-induced laminin functional changes may serve as a potential biomarker of alterations in the BBB. The 38 publications included evaluated neuroinflammation, BBB disruption, and laminin, and were assessed for quality and risk of bias (protocol registered in PROSPERO; CRD42020212547). We found that laminin may be a good indicator of BBB overall structural integrity, although changes in expression are dependent on the pathologic or experimental model used. In ischemic stroke, permanent vascular damage correlates with increased laminin expression (β and γ subunits), while transient damage correlates with reduced laminin expression (α subunits). Laminin was reduced in traumatic brain injury and cerebral hemorrhage studies but increased in multiple sclerosis and status epilepticus studies. Despite these observations, there is limited knowledge about the role played by different subunits or isoforms (such as 411 or 511) of laminin in maintaining structural architecture of the BBB under neuroinflammation. Further studies may clarify this aspect and the possibility of using laminin as a biomarker in different pathologies, which have alterations in BBB function in common.

## 1. Introduction

Neuroinflammation, defined as an inflammatory response within the brain or spinal cord [1], is a process triggered by different factors, including infection, autoimmunity processes, toxic metabolites, trauma, or bleeding [2]. The presence of neuroinflammation has been described in many diseases, including Parkinson’s disease (PD) [3], Alzheimer’s disease (AD) [4], stroke [5], multiple sclerosis (MS) [6], and epilepsy [7]. Despite the protective role of the inflammatory response, typically involved in pathogen elimination and tissue repair, chronically maintained inflammation can lead to homeostatic imbalance and tissue damage.

Neuroinflammation can appear in both acute and chronic central nervous system (CNS) responses [8]. Acute neuroinflammation involves the immediate activation of a defense response directed at tissue protection or reparation. During this phase, the nervous system activates immunological responses such as microglia, astrocytes, and vascular endothelium, promoting morphological changes, cell proliferation, and release of proinflammatory factors. Activated blood–brain barrier (BBB) endothelial cells may also attract immune cells from the periphery, which could compromise the inflammatory process through the induction of an immune response in the CNS. This event occurs when peripheral immune cells mobilize toward the perivascular zone, triggering the reactivity of brain endothelial cells, which leads to subsequent BBB leakage and a massive infiltration of immune cells [9,10]. If the BBB remains intact, and there is no induced infiltration of peripheral immune system cells, the acute neuroinflammatory process is limited only to the glial cell activation responses. In this scenario, the inflammation ceases once the pathogen or harmful stimulus is eliminated, and the reparative function of the glial cells begins [11]. In comparison, chronic neuroinflammation is characterized by the constant presence of harmful inflammatory stimuli, which affect the nervous system’s functioning, causing damage to the BBB and glial reactivity. Chronic low-level inflammation, as present in aging or posterior to traumatic brain injury (TBI), has been related to cognitive impairment and reduced plasticity, whereas chronic increased inflammatory activity, as present in neurodegenerative diseases, has been related to direct neuronal damage [1]. Therefore, sustained neuroinflammatory activity has been shown to be responsible for CNS malfunction, including BBB disruption and increased permeability.

BBB alterations are a common feature of CNS-related pathologies or injuries, mainly associated with a neuroinflammatory process leading to disruptive and nondisruptive changes. Disruptive alterations produce visible changes in the anatomy of the BBB, such as glia limitans collapse, astrocytopathy, glycocalyx degradation, and an increase in vesicular traffic [12]. In contrast, nondisruptive alterations involve astrocyte function modulation, cytokine synthesis, and changes in the expression of receptors or transporters. BBB alterations result in CNS vulnerability to peripheral molecules and signals, which may prolong neuroinflammation and further extend the BBB disruption. Therefore, it is essential to identify the different components of the BBB involved and how they are affected during neuroinflammatory pathological processes. This may have a clinical impact, as the comprehension of these factors may lead to a better characterization of the diseases and, possibly, improved patient diagnostic, prognostic, and therapeutic interventions [12].

Furthermore, a persistent neuroinflammatory state is associated with alterations in the expression of extracellular matrix (ECM) receptors and ligands, basal lamina proteins, membrane receptors, and tight junctions (TJ) of endothelial cells [13,14,15]. Cellular interactions with ECM proteins are fundamental to promote cell adhesion, neurite outgrowth, axon guidance, differentiation of neural progenitor cells, and angiogenesis [13,16,17].

Laminins are heterotrimeric glycoproteins that constitute the major non-collagenous component of basement membranes (BM) [18,19,20]. Several reports suggest the involvement of laminin in the regulation of the BBB function and in neuroinflammation [21,22,23,24,25,26,27]. There are 16 different isoforms that are composed of α, β, and γ polypeptide chains [18,20]. Laminin-511 (α5-β1-γ1) and laminin-411 (α4-β1-γ1) are differentially expressed in brain microvascular endothelial cells (BMECs) [24]. Laminin-511 has been reported to improve the BBB integrity by stabilizing the TJ and reducing leukocyte extravasation under neuroinflammatory conditions [28,29]. Moreover, several of the laminin subunits have been shown to be expressed in the rodent brain, including α1 to α5, β1, and γ1, while the β3 and γ1 chains have also been reported in sprouting neurons and rat astrocytes [30]. As a result, laminin activity has been proven to be compromised during neuroinflammatory processes. Due to its central role in cellular–ECM interactions and its changes in pathological conditions, laminin can be considered as a potential CNS biomarker. However, many gaps regarding laminin’s role in BBB integrity and neurovascular unit (NVU) function still remain [20].

The present systematic review collects, describes, and evaluates published evidence regarding the effects of neuroinflammation on laminin and the BBB in different animal models. This review is driven by our interest to evaluate laminin as a potential biomarker and is focused on analyzing functional changes in laminin induced by neuroinflammation, which could serve as a tool to assess BBB alterations.

## 2. Methods

The protocol for this systematic review was registered on 16 November 2020, in PROSPERO as PROSPERO 2020 CRD42020212547 (https://www.crd.york.ac.uk/prospero/display_record.php?ID=CRD42020212547 accessed on 13 June 2022), and it was carried out adhering to the Preferred Reporting Items for Systematic Reviews and Meta-Analyses (PRISMA) guidelines for reporting [31].

### 2.1. Search Strategy

The following databases were searched: PubMed, Scopus, and Web of Science (WoS). These databases were explored without date limits for articles published up to 16 September 2020. We used a combination of the following key search criteria (SC):

SC1: neuroinflammation, neuroinflammatory;
SC2: blood–brain barrier, central nervous system, permeability, disruption;
SC3: murine, rat, mouse, mice, animal;
SC4: laminin.

### 2.2. Eligibility Criteria

**Figure 1:**
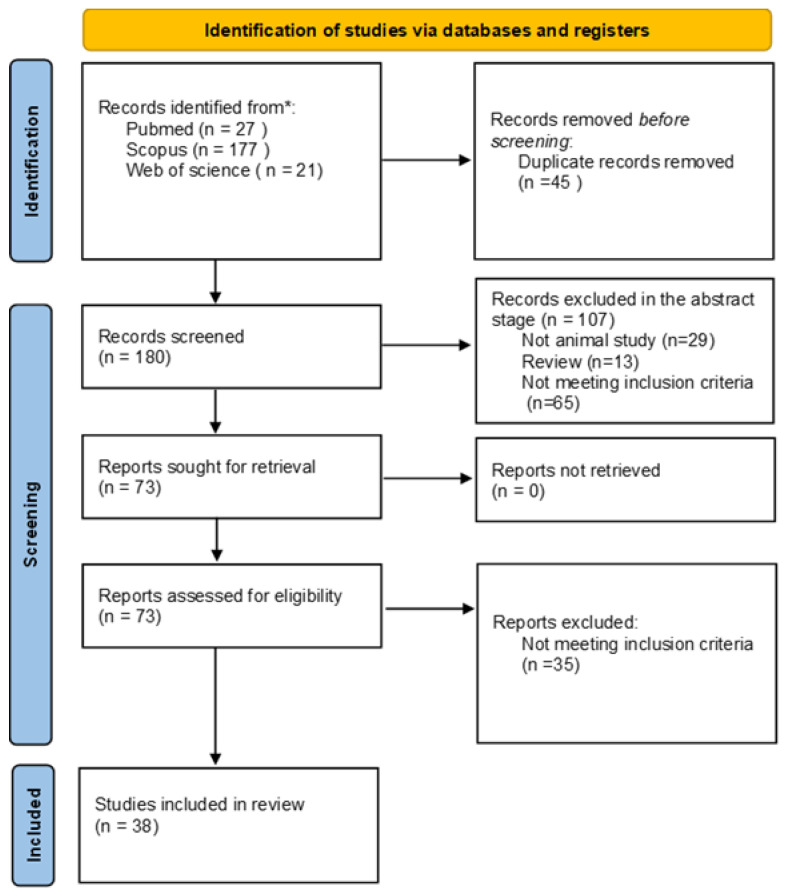
Flow diagram of study selection process. The flow diagram is divided into four phases: identification, screening, eligibility, and inclusion. * refers to scientific databases.

We included experimental studies written in English, which were fully available (full text), and performed in vivo (in animal models). No in vitro, in silico, or human studies were included. Moreover, literature reviews, conference abstracts, editorials, and theses were excluded. We considered the following interventions in the CNS to be included in the review: animal models of neurodegenerative diseases with neuroinflammatory effects, models of stroke, injections of proinflammatory substances such as lipopolysaccharide (LPS), or infections induced by pathogens; all of these, should have reported damage to the BBB due to neuroinflammation and measured changes in laminin. Therefore, studies included in this systematic review evaluated neuroinflammation, BBB disruption, and laminin. Articles were excluded if the interventions were performed only on one or two of the aforementioned criteria. Lastly, we excluded studies without a control group or any comparative criteria (see Figure 1).

### 2.3. Data Extraction

All studies that met the SC were obtained from the three databases explored. Duplicated or triplicated articles were excluded, and the remaining papers had the titles and abstracts screened in order to evaluate if eligibility criteria were met. Two different authors independently evaluated the titles and abstracts of all the selected papers and compared results. A third author was consulted if a discrepancy or doubt was present. Articles which met eligibility criteria were reviewed in full text and evaluated for biases using the Systematic Review Center for Laboratory Animal Experimentation (SYRCLE) risk of bias (RoB) tool, and for quality assessment using the Collaborative Approach to Meta Analyses and Review of Animal Data from Experimental Studies (CAMARADES) format, for in vivo studies, with slight modifications. The review and study selection processes are illustrated in a flow diagram (Figure 1), showing the number of all excluded studies, as well as the reasons for their exclusion.

The data were extracted according to (a) the type of experimental model, including neurological, neurodegenerative, cerebrovascular diseases, pharmacological interventions, physical interventions, or any other type of pathological model which induced brain parenchymal disorders, (b) alterations in BBB, (c) laminin measurements, (d) measurements of neuroinflammation markers, (e) time of the intervention and the method of neuroinflammation induction, (f) relationship among neuroinflammatory markers, BBB alterations, and changes in laminin, and (g) general aspects of experimental design (experimental groups, age and gender of animal, weight, etc.).

The in vivo results were organized according to the different experimental models, and evidence regarding laminin changes in the BBB due to neuroinflammation was analyzed.

### 2.4. Assessment of Methodological Bias and Quality of Studies

We used the SYRCLE RoB tool as described by Hooijmans et al. [32] to estimate the overall bias of selected studies in our review. Quality assessment was conducted with the CAMARADES format, with slight adjustments (www.camarades.info; www.dcn.ed.ac.uk/camarades/research.html#protocols accessed on 10 December 2020). Both the RoB and the quality assessment were performed by two different authors independently for each paper. In case of doubt or disagreement, a third author was consulted.

## 3. Results

A total of 180 articles were screened for the inclusion criteria. The search was performed in the scientific databases PubMed, Scopus, and Web of Science (WoS). After the abstract and title screening phase, 76 studies were eligible for full-text analysis. Finally, after the full-text screening, 38 articles were included for qualitative analysis (Figure 1). These 38 original studies were categorized by the CNS disease/experimental model in Table 1, which also includes changes in laminin, cellular and molecular neuroinflammatory characteristics, and BBB alterations.

The distribution of the studies, according to the pathological model, was as follows: eight studies on cerebral ischemia, mostly induced by cerebral artery occlusion [30,33,34,35,36,37,38,39], five studies in cerebral hemorrhage induced by different methods [40,41,42,43,44], five studies on TBI, mostly by controlled cortical impact (CCI) [45,46,47,48,49], three studies combining cerebral ischemia and AD [50,51,52], three studies on aging [53] or AD models, using either amyloid-β (Aβ) [54] or transgenic animals [55], two studies on the experimental autoimmune encephalomyelitis (EAE) animal model for MS, induced by immunization with myelin oligodendrocyte glycoprotein (MOG) [56,57], three studies on status epilepticus (SE), induced with lithium chloride (LiCl)/pilocarpine or kainic acid (KA) treatment [58,59,60], five studies using diverse pharmacological or toxicological agents such as 3,4-methylenedioxymethamphetamine (MDMA), methamphetamine (METH), 3-chloro-1,2-propanediol, LPS, and glutaric acid (GA) [61,62,63,64,65], two studies involving pathogen-induced diseases, one in toxoplasmosis and another in human immunodeficiency virus (HIV) [66,67], one study which induced cognitive dysfunction by laparotomy [68], and one study which used the platelet-derived growth factor receptor β (PDGFRβ)^+/−^ knockout model [69]. The three studies that combined stroke and AD were assigned to an independent category rather than being included in either stroke or AD categories. This distinction was due to methodological differences in the experimental model used in these studies, which characterized both diseases in the animals instead of only one.

A diverse range of animal models were used in the included studies. Fourteen studies were conducted in Sprague-Dawley rats, three in Wistar rats, one in Fisher F344 rats, one in Dark Agouti rats, 10 in C57BL/6J mice, one in CD1 mice, two in BALB/c mice, one in 3xTg mice (which contains three mutations associated with familial AD: APP Swedish, MAPT P301L, and PSEN1 M146V), one in EFAD mice (which express human apolipoproteins E (APOE) 3 or APOE4, and overproduce human Aβ via the expression of five familial AD (5xFAD) mutations), one in the β4 integrin flox/flox transgenic mice, one in C57BL/10 RIII H2 mice, one in Swiss Webster mice, one in PDGFRβ^+/−^ mice, two in matrix metallopeptidase 9 (MMP-9) knockout mice, one in MMP-3 knockout mice, one in cyclooxygenase-2 (COX-2) knockout mice, one in agrin knockout mice, one in NG2 knockout mice, and one in F7 mice. According to sex distribution, 16 studies used male rats, but only one used female rats; 10 studies used male mice, three used female mice, two mouse studies were performed in both sexes, and six failed to report the sex of the animals.

**Table 1:** General characteristics of the included studies.

	Author (Year)	CNS Disease Experimental Model	Animal Details	Animal Groups		Main Outcome
Stroke	McColl BW et al. (2008) [37]	**Stroke:** tMCAO for 30 min (side not specified). Systemic inflammation was induced by injection of recombinant IL-1β.	C57BL/6J mice (sex not specified); 25–30 g; 10–12-weeks	**EG: a)** tMCAO (vehicle); **b)** tMCAO + IL-1β; *n* is not reported	**Control:** Sham-operated; *n* is not reported	In temporary ischemia under neuroinflammation, no laminin changes were found in the vessels. However, the BBB showed decreased TJ proteins (claudin-5 and occludin).
	Ji K, Tsirka SE. (2012) [30]	**Stroke:** Unilateral (side not specified) pMCAO using a small 6–0 siliconized monofilament.	**a)** C57BL/6 adult mice (sex not specified); **b)** C57BL/6 COX-2 KO	**EG: a)** WT + pMCAO; **b)** COX-2 KO + pMCAO; *n* is not reported	**Control:** Sham-operated + not ischemic side; *n* is not reported	In permanent ischemia, increased laminin α2, β3, and γ1 subunits were found, together with increased microglia activation and immune cell infiltration (CD45^+^). The BBB permeability was augmented with increased angiogenesis (VEGF).
	Steiner E et al. (2012) [38]	**Stroke:** Left tMCAO for 30 min, followed by ischemia/reperfusion (I/R) protocols: I/R: 30 min/12, 24, and 48 h.	**a)** C57BL/6 male mice, 8 to 12 weeks; **b)** agrin KO male mice (c-magB8//agrn^−/−^), 10–14 weeks; **c)** transgenic agrin male mice (c-magB8//agrn^+/+^), 10–14 weeks	**EG: a)** WT + tMCAO; **b)** c-magB8//agrn^−/−^ + tMCAO; **c)** c-magB8//agrn^+/+^ + tMCAO; *n* is not reported	**Control:** Sham-operated; *n* is not reported	In temporary ischemia, laminin α2 and α4 subunits decreased. No changes in BBB were found, and neuroinflammation was not established. Astrocytes decreased expression of AQP4.
	Lee JY et al. (2014) [36]	**Stroke:** Transient bilateral common carotid artery occlusion for 15 min.	CD1 adult male mice; 33–37 g; 8 weeks	**EG:** Stroke group; *n* is not reported	**Control:** Sham-operated; *n* is not reported	In temporary ischemia, laminin α4 subunit decreased. BBB permeability increased with decreased occludin expression. Neuroinflammation was established with increased inflammatory markers, microglial activation, and astrocytic reactivity.
	Boroujerdi A et al. (2015) [33]	**Chronic hypoxia model:** MMP-9 KO and C57BL/6 mice were housed in a hypoxic chamber (8% oxygen) for a period of 14 days. Mice were then removed and maintained for 7 to 14 days in normal oxygen levels.	**a)** C57BL/6 mice (sex not specified), 8–10 weeks; **b)** MMP-9 KO 8–10 weeks	**EG: a)** MMP-9 KO + hypoxia and post hypoxia; **b)** WT mice + hypoxia and post hypoxia; *n* is not reported	**Control:** Normoxic mice; *n* is not reported	In chronic ischemia, vascular laminin decreased due to increased MMP-9 levels. Chronic neuroinflammation induced claudin-5 decrease.
	Cisbani G et al. (2018) [34]	**Stroke:** pMCAO by unilateral (right side) application of ferric chloride. Mice were sacrificed 48 h and 7 days post surgery.	**a)** C57BL/6 adult male mice, 6 months; **b)** CCR2 KO adult male mice, 6 months (B6.129S4-Ccr2tm1Ifc/J); **c)** CX3CR1 KO adult male mice, 6 months (B6.129P-Cx3cr1tm1Litt/J)	**EG: a)** CCR2 KO + pMCAO; **b)** CX3CR1 KO + pMCAO; *n* = 10–20 mice per group	**Control:** WT + pMCAO; *n* = 10–20 mice	In permanent ischemia, laminin increased and CD45^+^ infiltration decreased.
	Wang J et al. (2018) [39]	**Stroke:** Right tMCAO for 60 min.	C57BL/6 male mice; 25–30 g; 12–14 weeks	**EG:** tMCAO; *n* = 82	**Control:** Sham-operated treated with saline; *n* = 72	In temporary ischemia, laminin decreased. The BBB permeability increased through a decrease in ZO-1. Neuroinflammatory markers and microglia were increased.
	Kim DY et al. (2020) [35]	**Stroke:** Left tMCAO for 90 min.	C57BL/6J male mice; 8–10 weeks	**EG:** C57BL/6J + tMCAO; *n* = 5	**Control:** Sham-operated; *n* = 5	In temporary ischemia, laminin decreased. The BBB permeability increased. Astrocytes increased GFAP and AQP4.
Cerebral Hemorrhage	Suzuki H et al. (2010) [41]	**SAH:** Perforation of anterior and middle cerebral artery bifurcation.	Sprague-Dawley rats; adult male; 300–370 g	**EG:** SAH rats treated with saline; *n* = 45	**Control:** Sham-operated rats were treated with saline; *n* = 23	Hemorrhage induced laminin decrease. The BBB permeability increased through a decrease in ZO-1. Neuroinflammatory markers were increased (metalloproteinases and cytokines).
	Zhang XS et al. (2015) [44]	**SAH:** A total of 0.3 mL of non-heparinized fresh autologous arterial blood from the femoral artery was slowly injected into the prechiasmatic cistern.	Sprague-Dawley rats; adult male; 250 and 300 g	**EG:** SAH; *n* = 36	**Control:** Animals were injected with 0.3 mL of saline; *n* = 36	Hemorrhage induced laminin decrease. The BBB permeability increased, together with augmented microglia activation and infiltration of neutrophils. Neuroinflammatory markers were increased (metalloproteinases and cytokines).
	Zeng J et al. (2018) [43]	**CMH:** 1 mg/kg dose of LPS derived from *Salmonella typhimuriu* was injected i.p. into rats at 3 different timepoints (0, 6, and 24 h), which induced CMH features 7 days after the injections.	Sprague-Dawley rats; male; 280 ± 20 g; aged 10 weeks	**EG:** LPS-treated rats at 0, 6, and 24 h; *n* = 20	**Control:** 1 mg/kg dose of PBS i.p. injection at 0, 6, and 24 h; *n* = 20	Hemorrhage induced laminin decrease and BBB permeability increase through a decrease in ZO-1. Neuroinflammation was established through increased microglial activation, together with increased astrocytic GFAP.
	Gautam J et al. (2020) [40]	**Intracerebral hemorrhage**: Induced with injected collagenase type VII-S (0.15 U in 0.7 μL saline) into the striatum over 5 min.	C57BL/6J mice: both sexes; 6–8 weeks old	**EG:** Laminin-γ1 flox/flox mice were crossed with the PDGFRβ-Cre^+^ line to generate PKO (laminin-γ1 flox/flox:Pdgfrβ-Cre^+^) mice; *n* is not reported	**Control:** Sham controls (striatum injected with saline); *n* is not reported	Hemorrhage induced laminin decrease and BBB permeability increase. Neuroinflammation was established through increased microglial activation, together with increased astrocytic GFAP.
	Wang F et al. (2020) [42]	**SAH:** Blood injection into the pre-chiasmatic cistern.	Sprague-Dawley rats; male; 300–350 g	**EG:** SAH group; *n* = 10	**Control:** Sham group, injected normal saline into the pre-chiasmatic cistern; *n* = 15	Hemorrhage induced laminin α2 subunit decrease. BBB permeability was augmented through a decrease in TJ proteins (occludin and ZO-1). Astrocytes GFAP was increased.
Traumatic Brain Injury	Gursoy-Ozdemir Y et al. (2004) [45]	**CSD:** Dura mater was opened and recording electrodes were placed over both hemispheres. CSD was initiated by pinprick with a 30-gauge needle.	Sprague-Dawley rats; male; 250–300 g or MMP-9-null male mice and age-matched CD1 male mice; 25 g	**EG:** CSD induction; *n* is not reported	**Control:** Operated but no CSD induction; *n* is not reported	Traumatic injury induced laminin decrease. The BBB permeability increased through a decrease in ZO-1. MMP-9, as a marker of neuroinflammation, was increased.
	Reyes R et al. (2009) [47]	**Thermal injury/burn encephalopathy:** Rats were submerged horizontally for 6 s, supine, in either 100 °C water for the thermal injury group or 37.5 °C for the control group.	Sprague-Dawley rats; male; 260–280 g	**EG:** Thermal injury groups; *n* = 16	**Control:** No thermal injury; *n* = 8	Traumatic injury induced laminin decrease. The BBB permeability augmented together with an increase in MMP-9.
	Higashida T et al. (2011) [46]	**TBI:** To produce TBI, a modified Marmarou force impact model was used.	Sprague-Dawley rats; male; 400–425 g	**EG:** TBI group; *n* = 18	**Control:** Sham-operated (not subjected to the weight drop); *n* = 18	Traumatic injury induced laminin decrease. The BBB permeability increased through a decrease in TJ proteins (occludin and ZO-1). MMP-9, as a marker of neuroinflammation, was increased, together with increased astrocytic AQP4.
	Tao X et al. (2015) [48]	**TBI:** CCI in mice was produced using PCI3000 PinPoint Precision Cortical Impactor.	BALB/c mice; male; 20–25 g; 12–13 weeks of age	**EG:** Mice with CCI; *n* = 8	**Control:** Sham-operated (without percussion); *n* = 8	Traumatic injury induced laminin decrease. The BBB permeability increased through a decrease in occludin. Neuroinflammatory markers increased (metalloproteinases and cytokines), together with neutrophil infiltration.
	Tao XG et al. (2017) [49]	**TBI:** CCI in mice was produced using PCI3000 PinPoint Precision Cortical Impactor.	BALB/c mice; male; 25–30 g; 12–13 weeks of age	**EG:** Mice with CCI; *n* = 42	**Control:** Sham-operated (without percussion); *n* = 24	Traumatic injury induced laminin decrease. The BBB permeability increased through a decrease in occludin. Neuroinflammatory markers increased (metalloproteinases, cytokines, and ICAM-1), together with changes in astrocyte morphology.
Cerebral Ischemia and Alzheimer’s Disease	Hawkes CA et al. (2013) [52]	**Stroke:** Right side pMCAO using a small 6–0 siliconized monofilament. **AD:** 3xTransgenic mice harbored 2 mutant human transgenes (APP Swedish mutation and tauP301L)—driven by neuron-specific Thy1-regulatory elements—added by the homozygous knock-in construct presenilin-1M146V	**a)** Sv129/B6 (WT) 3 and 12-month, both sexes; **b)** 3xTransgenic mice, 3 and 12 months, both sexes	**EG: a)** Stroke (WT) of 3 (*n* = 14) and 12 month old (*n* = 6); **b)** Stroke (3xTransgenic) of 3 (*n* = 9) and 12 month old (*n* = 9)	**Control: a)** Sv129/B6 mice, *n* not reported; **b)** the contralateral side of the brain in both experimental groups, *n* not reported	Permanent ischemia in AD mice induced laminin increase. Microglia showed activation and astrocytic AQP4 decreased.
	Amtul Z et al. (2018) [50]	**Ischemia:** Induced by ET1 injected into the right striatum, causing permanent damage. **AD:** Model of Aβ toxicity induced by bilateral injection of Aβ25–35 into the lateral ventricles.	Wistar rats; male; 250 to 310 g	**EG: a)** ET1 group, *n* = 6; **b)** AD, *n* = 6; **c)** AD + ET1 group, *n* = 6	**Control:** Sham-operated (saline injections); *n* = 6	Permanent ischemia in AD rats induced laminin increase. BBB permeability was augmented, together with increased infiltration of immune cells and MMP-9 activity. Microglia showed activation and astrocytic GFAP and AQP4 were increased.
	Amtul Z et al. (2019) [51]	**Ischemia:** Induced by ET1 injected into the right striatum, causing permanent damage. **AD:** Model of Aβ toxicity induced by bilateral injection of Aβ25–35 into the lateral ventricles.	Wistar rats; male; 240–310 g	**EG: a)** ET1 group, *n* = 6; **b)** AD, *n* = 6; **c)** AD + ET1 group, *n* = 6	**Control:** Sham-operated (saline injections); *n* = 6	Permanent ischemia in AD rats induced laminin increase. BBB permeability was augmented, together with increased infiltration of immune cells and MMP-9 activity. Microglia showed activation, and astrocytic GFAP and AQP4 were increased.
Alzheimer’s Disease and Aging	Campbell SJ et al. (2007) [53]	**Age + neuroinflammatory induction:** IL-1β (1 ng), TNF-α (1 μg), or sterile endotoxin free saline was microinjected into the striatum.	**a)** Wistar rats, sex not specified, 40–50 g, juvenile 3 weeks old; **b)** Wistar rats, sex not specified, 200 g, young adult 2 months old; **c)** Wistar rats, >450 g, aged 18 months old	**EG: a)** Juvenile + IL-1β, *n* = 3 or TNF-α, *n* = 3; **b)** young adult + IL-1β, *n* = 3 or TNF-α, *n* = 3; **c)** aged + IL-1β, *n* = 3 or TNF-α, *n* = 3	**Control:** Mice injected with vehicle: **a)** juvenile, *n* = 3; **b)** young adult, *n* = 3; **c)** aged, *n* = 3	Neuroinflammation in aged rats induced laminin decrease. BBB permeability was augmented, together with a decrease in claudin-1. Microglia showed activation, together with an increase in proinflammatory cytokines from infiltrating immune cells.
	Ryu JK, McLarnon JG. (2008) [54]	**AD:** Peptides (full-length Aβ1–42 or reverse peptide Aβ42–1) were slowly injected into the dentate gyrus of hippocampus for a duration of 7 days.	Sprague-Dawley rats; male; 280–300 g	**EG: a)** Full-length Aβ1–42, *n* = 15; **b)** reverse peptide Aβ42–1, *n* = 15	**Control:** Injected with carboxymethylcellulose in PBS; *n* = 15	Rat AD model induced laminin increase. BBB permeability was increased. Microglia showed activation, together with an increase in proinflammatory cytokines. Astrocytic GFAP was increased.
	Marottoli FM et al. (2017) [55]	**AD:** EFAD mice were generated by crossing ApoE-targeted replacement mice with female 5xFAD mice. EFAD mice were administered PBS or LPS via intraperitoneal injection (0.5 mg/kg/week) from 4 to 6 months. A final LPS treatment was administered the day before sacrifice for nine total injections.	EFAD male mice; 4–6 months old	**EG: a)** E3FAD − LPS, *n* = 7; **b)** E3FAD + LPS, *n* = 7; **c)** E4FAD − LPS, *n* = 11; **d)** E4FAD + LPS, *n* = 12	**Control: a)** E3FAD − PBS, *n* = 9; **b)** E3FAD + PBS, *n* = 7; **c)** E4FAD − PBS, *n* = 10; **d)** E4FAD + PBS, *n* = 9	Transgenic mice AD model induced laminin decrease. BBB permeability was increased. Neuroinflammatory markers and cytokines were increased.
Multiple Sclerosis	Welser JV et al. (2017) [57]	**EAE:** induction was by immunization with MOG33–35 peptide emulsified in complete Freud’s adjuvant (CFA) containing *Mycobacterium tuberculosis* and an additional injection of pertussis toxin.	**a)** The β4 integrin flox/flox transgenic mice; **b)** WT littermate control (β4flox/WT). All strains were backcrossed >10 times onto the C57BL/6b; 8–10 week old female mice.	**EG:** β4 integrin flox/flox transgenic mice+ MOG33–35 peptide emulsified in complete Freud’s adjuvant; *n* = 4	**Control:** WT mice received complete Freud’s adjuvant containing no MOG peptide; *n* = 4	MS model induced laminin increase. The BBB permeability increased through a decrease in TJ proteins (claudin-5 and ZO-1). Microglia showed activation, and immune cell infiltration was present.
	Girolamo F et al. (2019) [56]	**EAE:** Chronic EAE was induced by active immunization with MOG peptide spanning amino acids 35–55 in female WT and NG2 KO mice.	WT C57BL/6J mice and NG2 KO mice; female; 18.5 g ± 1.5 g; 8 weeks old	**EG: a)** WT + EAE, *n* = 4–6; **b)** NG2 KO + EAE group, *n* = 4–6	**Control:** No EAE; **a)** WT, *n* = 4–6; **b)** NG2 KO, *n* = 4–6	MS model induced laminin increase. The BBB permeability increased through a decrease in TJ proteins (claudin-5 and occludin).
Status Epilepticus	Sarkar S, Schmued L. (2010) [60]	**Kainic acid:** The first group received kainic acid (10 mg/kg; i.p.), the second group received 3-NPA (3 doses of 20 mg/kg); s.c. one day interval.	Sprague-Dawley rats; male; 400–450 g	**EG: a)** Kainic acid, *n* = 5; **b)** 3-NPA, *n* = 5	**Control:** Received only saline (2 groups); *n* = 5 per group	Laminin increased. The permeability of BBB increased, and there was microglia activation.
	Kim YJ et al. (2014) [58]	**Vasogenic edema formation induced by SE:** Animals were given LiCl (127 mg/kg i.p.) 20 h, and then they were treated with pilocarpine (25 mg/kg, i.p.) 30 min after scopolamine butylbromide (2 mg/kg, i.p.).	Sprague-Dawley rats; male; 7 weeks old	**EG:** SE (12 h, 1 day, 3 days, 4 days, 1 week, and 4 weeks after SE); *n* = 7 for each timepoint	**Control:** non-SE; *n* = 7	SE induced laminin increase. BBB permeability was increased. Astrocytic GFAP was increased.
	Park H et al. (2019) [59]	**Vasogenic edema formation induced by SE:** Rats were pretreated with i.p. injection of LiCl (127 mg/kg i.p) 24 h before the pilocarpine treatment. Pilocarpine was injected i.p. (30 mg/kg) for 20 min after atropine methylbromide (5 mg/kg i.p.).	Sprague-Dawley rats; male; 7 weeks old	**EG:** SE; *n* is not reported	**Control:** Non-SE saline instead of pilocarpine; *n* is not reported	SE induced laminin increase. BBB permeability was increased, together with an increase in VEGF. Astrocytic AQP4 was decreased.
Pharmacological and Toxicological Interventions	Gurney KJ et al. (2006) [61]	**Neuroinflammation model**: Induced by intracerebral injections of LPS (15 ng in 1 μL of saline) into the caudate/putamen.	**a)** WT C57Bl/10 RIII H2 mice, male, 25–30 g; **b)** MMP-3 KO mice, 25–30 g	**EG: a)** WT + LPS, *n* = 35; **b)** MMP-3 KO + LPS, *n* = 35	**Control: a)** Sham, *n* = 35; **b)** control craniotomy + saline, *n* = 35	LPS injection induced laminin decrease. The BBB permeability increased through a decrease in TJ proteins (claudin-5 and occludin). Neuroinflammation induced an increase in MMP-3 and MMP-9, together with activation of pericytes.
	Urrutia A et al. (2013) [64]	**METH:** Animals received 3 injections of (+)-METH (4 mg/kg, i.p.) in striatal at 3 h intervals and were sacrificed 1, 3, or 24 h after drug dosing.	C57BL/6J mice; male; 25–30 g	**EG:** METH; *n* is not reported	**Control:** Saline; *n* is not reported	METH injection induced laminin decrease. The BBB was increased. Neuroinflammation induced an increase in MMP-9.
	Willis CL et al. (2013) [65]	**3-chloro-1,2-propanediol:** Single i.p. dose of 140 mg/kg (*S*)-(+)-3-chloro-1,2-propanediol.	Fisher F344 rats; male; 180–220 g	**EG:** Fisher F344 rats + (*S*)-(+)-3-chloro-1,2-propanediol (1, 2, 3, 4, 5, 6, and 8 days); *n* is not reported	**Control:** Vehicle injected animals (0 days); *n* is not reported	3-Chloro-1,2-propanediol injection induced laminin increase around the vascular endothelium. The BBB permeability increased through a decrease in TJ proteins (claudin-5 and occludin). Neuroinflammation induced an increase in macrophage infiltration, and astrocytic GFAP was increased.
	Isasi E et al. (2014) [62]	**GA:** Rat pups were injected at postnatal day 0 with GA (1 μmol/g body weight, pH 7.4) or PBS (1 μmol/g body weight; 10 mM, pH 7.4) into the cisterna magna to reproduce a GA-like encephalopathic crisis.	Sprague-Dawley rats (sex not specified); 12 and 24 h postnatal	**EG:** GA; *n* not reported	**Control:** PBS; *n* not reported	GA injection induced laminin decrease. BBB permeability was increased. Astrocytic AQP4 was decreased.
	Rubio-Araiz A et al. (2014) [63]	**MDMA-induced model of neuroinflammation:** MDMA was given at the dose of 10 mg/kg (i.p.). Animals were sacrificed 1, 3, 6, or 24 h later.	Dark Agouti rats; male; 175–200 g	**EG:** MDMA; *n* is not reported	**Control:** Saline; *n* is not reported	MDMA injection induced laminin decrease. BBB permeability was increased. Neuroinflammation induced an increase in MMP-3 and MMP-9.
Pathogen Induced Disease	Louboutin JP et al. (2010) [67]	**HIV-1:** gp120 injection (500 ng of gp120 in 1 μL of saline) in the caudate-putamen.	Sprague-Dawley rats; female; 300–350 g	**EG:** gp120; *n* = 128	**Control:** Saline; *n* = 93	HIV-1 induced laminin decrease. The BBB permeability increased through a decrease in TJ proteins (claudin-5). Neuroinflammation induced an increase in MMP-2 and MMP-9.
	Estato V et al. (2018) [66]	**Toxoplasmosis:** Swiss Webster mice were infected i.p. with 50 *T. gondii* tissue cysts in a final volume of 100 μL in PBS.	Swiss Webster mice; female; 40 days old	**EG:** Infection group treated with the cysts; *n* not reported	**Control:** injected with equivalent dose of PBS; *n* not reported	Toxoplasmosis induced laminin decrease. BBB permeability was increased. Microglia showed activation and leukocytes infiltration was present.
Others	Bell RD et al. (2010) [69]	**Pdgfrβ^+/−^ and F7 mice:** In Pdgfrβ^+/−^ mice, a PGKneobpA expression cassette was used to replace the gene coding for the signal peptide to the second immunoglobulin domain of PDGFRβ. F7 mice were generated by point mutations that disrupted the following residues: 578 (Src), 715 (Grb2), 739 and 750 (PI3K), 770 (RasGAP), and 1008 (SHP-2), by changing the tyrosine to phenylalanine.	Pdgfrβ^+/−^ (sex not specified), 6 to 8 months old and F7 mice, 14 to 16 months old (sex not specified)	**EG: a)** Pdgfrβ^+/−^ mice, *n* is not reported; **b)** F7 mice, *n* is not reported	**Control:** Pdgfrβ^+/+^ mice; *n* is not reported	Pdgfrβ^+/−^ mice showed laminin decrease. The BBB permeability increased through a decrease in TJ proteins (claudin-5, occludin, and ZO-1). Neuroinflammation was present, together with an increase in cytokines and ICAM-1.
	Li Z et al. (2016) [68]	**Postoperative cognitive dysfunction:** Rats received laparotomy under isoflurane anesthesia.	Sprague–Dawley rats; male; 20 months-old	**EG:** Surgery group; *n* = 52	**Control:** Sham (anesthesia only); *n* = 28	Postoperative cognitive dysfunction induced laminin increase. The BBB permeability increased through a decrease in TJ proteins (occludin and ZO-1). Neuroinflammation was present, together with an increase in cytokines and MMPs.

This table summarizes the experimental models used in each paper, together with the details of the animals used, including species, sex, age, and weight, and the characteristics of each experimental group (EG) and controls, including *n* (numbers). Abbreviations: 3-nitropropionic acid (3-NPA); Alzheimer’s disease (AD); amyloid beta (Aβ); apolipoprotein E (ApoE); C–C chemokine receptor type 2 (CCR2); classical cerebral microhemorrhages (CMH); controlled cortical impact (CCI); cortical spreading depression (CSD); cyclooxygenase-2 (COX2); CX3C chemokine receptor 1 (CX3CR1); experimental autoimmune encephalomyelitis (EAE); endothelin-1 (ET1); glutaric acid (GA); interleukin (IL); lipopolysaccharide (LPS); lithium chloride (LiCl); matrix metalloproteinase (MMP); 3,4-methylenedioxymethamphetamine (MDMA); methamphetamine (METH); middle cerebral artery occlusion (MCAO); multiple sclerosis (MS); myelin oligodendrocyte glycoprotein (MOG); neuron/glia antigen 2 (NG2); permanent MCAO (pMCAO); phosphate-buffered saline (PBS); platelet-derived growth factor receptor beta (Pdgfrβ); status epilepticus (SE); subarachnoid hemorrhage (SAH); tight junction (TJ); traumatic brain injury (TBI); transient MCAO (tMCAO); tumor necrosis factor (TNF); wildtype (WT).

### 3.1. Study Quality and Risk of Bias

**Figure 2:**
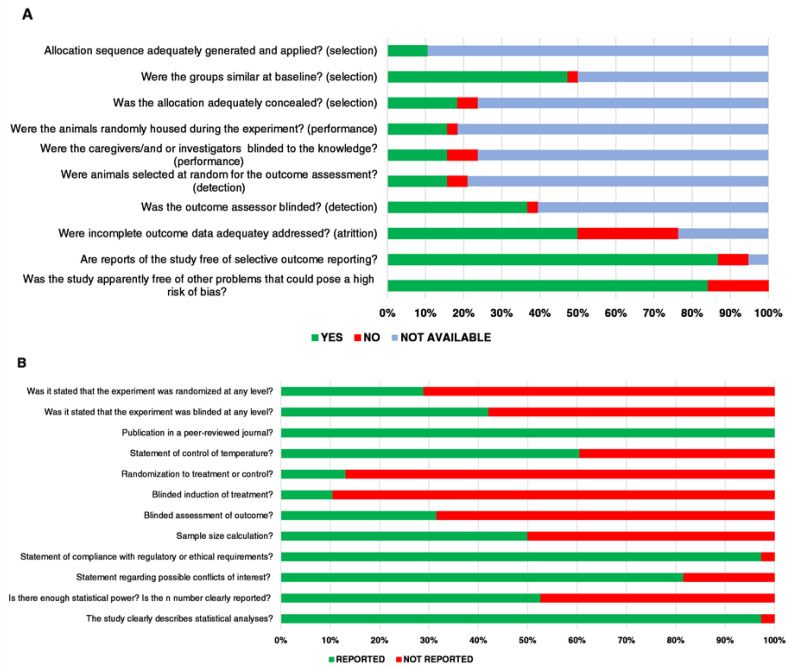
Risk of bias and quality assessment. The SYRCLE RoB Tool was used for assessing risk of bias for the included studies and assessment of study quality based on CAMARADES format. (**A**) Risk of bias assessment. These 10 items assessed selection, performance, detection, and attrition biases. The responses were “yes” for low risk of bias, “no” for high risk of bias, and “not available (N/A)” for unclear or unreported risk. (**B**) Study quality assessment. These 12 items assessed the overall quality of the included studies, stating if they were performed (“reported”) or not (“not reported”).

The assessment of study quality and RoB for all the included studies is presented in Figure 2. Individual assessments of all the studies for RoB and study quality are presented in Table 2 and Table 3, respectively. For many items, the quality of the report presented by the included studies was found to be poor, as too much RoB was obtained. The selection bias assessment demonstrated that only 11% of the studies generated allocation sequence adequately, while fewer than 47% of the studies reported that their groups were similar at the baseline. Furthermore, 18% of the articles detailed an adequately concealed allocation and random selection of the animals. Concerning the risk of performance bias, only 16% of studies stated that they randomly housed the animals during the experiment, and that the investigators were blinded during the experiments. Regarding the risk of detection bias, 37% of the studies reported that the investigator was blinded during the outcome assessment. However, only 16% clearly mentioned that animals were randomly selected for outcome assessment. In addition, 50% of the studies adequately addressed incomplete data outcomes to minimize the risk of attrition bias, and 87% of the study reports were free of selective outcome reporting.

Approximately 97% of our included studies described statistical analyses, but only 53% of the studies had a large enough sample size (*n* number) to obtain statistical power (47% of the included studies reported a prior sample size calculation). Almost all (97%) of the included articles mentioned that their experiments had ethical approval by an institutional animal care and use committee (IACUC), while only 18% did not report any information about conflicts of interest. Furthermore, 29% of experiments had randomization at any level, but only 13% of studies reported randomization to treatment or control, while 42% of experiments mentioned blinding at any level. Lastly, 61% of the studies included temperature control statements, and all the articles were published in a peer-reviewed journal.

Changes in laminin, BBB, BBB permeability, and neuroinflammation for each paper are presented in Table 4, while changes in cellular (including microglia, NVU cells, and immune system cells and markers) and ECM components for each paper are presented in Table 5. The column indicating laminin changes is included in both tables to facilitate reading and interpretation of the results.

### 3.2. Stroke

Stroke occurs when a brain blood vessel is either occluded/collapsed (ischemic stroke) or damaged enough to allow bleeding (hemorrhagic stroke). During ischemic stroke (the most common type of stroke representing 85% of cases), the stationary blood promotes an inflammatory response, involving the activation of intravascular leukocytes, the appearance of oxidative stress and excitotoxicity, and the release of proinflammatory cytokines, which have the potential to increase cellular damage [5,70]. Stroke is accompanied by neuroinflammation, which induces an early remodeling of the ECM, affecting the expression of laminin [30]. As laminin is important for BBB integrity [71], changes in this protein may reveal important pathological features of stroke.

Eight studies conducted on different stroke or ischemia animal models met the inclusion criteria and were incorporated in this systematic review. Two articles used a permanent middle cerebral artery occlusion (MCAO) model (one with direct injury [30] and the other with ferric chloride [34]), while five induced transient ischemia (four in the middle cerebral artery [35,37,38,39] and one in both common carotid arteries [36]). Transient ischemia times ranged from 15 min to 90 min. Lastly, one study induced chronic hypoxia after placing the animals for 14 days in a hypoxic chamber with a controlled oxygen concentration of 8% [33]. Seven studies used C57BL/6 mice, while one used CD1 mice. All studies used male animals, except three where it was not specified.

Changes in laminin were evaluated in the different ischemia and hypoxia models studied. In this aspect, both laminin expression or function were found to be reduced in all the studies which used transient ischemia or hypoxia experimental models, while, in both studies of permanent MCAO, laminin expression was increased, regardless of the timepoint examined. Likewise, two studies that used the transient ischemia model reported that the changes in laminin were in the α4 subunit, while one study that used permanent MCAO found that the changes were in the α2, β3, and γ1 laminin subunits.

The paragraphs below describe changes in laminin, together with changes in relevant inflammatory and BBB markers present in stroke studies with a permanent occlusion. Laminin was found to be increased from 6 to 12 h after a permanent MCAO stroke model, established with a siliconized monofilament [30]. This study used both wildtype mice and COX-2 knockouts. The laminin increase was only found on the wildtype mice with stroke, as the COX-2 knockout mice presented no changes in laminin after stroke induction. The three laminin subunits, α (α2, α4, and α5), β (β1 and β3), and γ (γ1 and γ2), were also examined, finding a strong increase in β3 and γ1 subunit expression, and a lesser increase in α2 subunit expression, following the ischemic injury. Furthermore, fibrinogen, fibronectin, and collagen IV were increased, and these changes were proportional to the infiltration of leukocytes and microglial activation. This study also examined BBB permeability markers such as CD45 and vascular endothelial growth factor (VEGF), reporting an increase in CD45 observed at 1 h, and between 24 and 48 h (but not at 3 to 6 h), while VEGF showed an increase from 6 to 12 h post ischemia. The inflammatory markers CD14 and TLR9 showed an increase in expression at 6 h, but not at 12 h. Laminin was also found to be increased in the study that established permanent MCAO using ferric chloride [34]. In this study, laminin was increased 7 days after the occlusion in all the stroke groups (wildtype, and in CX3CR1 and in CCR2 knockouts). Both the percentage of area occupied by laminin staining and the density of small laminin-positive vessels were increased in CX3CR1 knockout mice compared with wildtype mice. No differences between wildtype and CCR2 knockout mice were observed. The inflammatory markers TREM2 and TLR2 were also evaluated. TREM2 was found to be significantly increased in CCR2 and CX3CR1 knockout mice 7 days after the occlusion, but not in wildtype mice. No changes were observed in TLR2 or CD68 in any of the groups. Peripheral cell infiltration was studied, finding that leukocytes identified with the marker CD45 were higher in wildtype and CX3CR1 knockout mice than in CCR2 knockout mice at 48 h post surgery. Neutrophils, positive for the marker 7/4, were also present in the injured area only at 48 h post ischemia, but not afterward. This study was limited by a lack of sham-operated controls. The results showed that, in permanent MCAO stroke models, laminin was increased from 6 h up to 7 days after the injury. Although only one study examined them, the changes in laminin may be represented by an increase in the expression of subunits β3, γ1, and α2. Moreover, laminin increase seems to involve inflammatory components, as COX-2 knockout failed to augment it.

Several studies examined the effects of transient interruption of blood flow or chronic hypoxia in animal models. Only one experiment affected bilateral blood flow in both carotids, while the others were unilateral (side: two left, two right, one not mentioned) in the middle cerebral artery. The bilateral carotid experiment was also the one with the shortest blood interruption time (15 min), while the other studies had interruption times of 30 min (two studies), 60 min, and 90 min. Similarly, only one study used a chronic hypoxia model, placing mice in a chamber with 8% oxygen for 14 days, followed by 7 or 14 days at normal oxygen levels. Although low, this level of oxygen is not enough to cause permanent pathological changes in the animals. The study by McColl and colleagues [37] reported preservation of vessel-associated laminin, both in the transient occlusion group and in the transient occlusion group challenged with IL-1β, but they did observe loss of neuronal laminin 24 h after the MCAO.

The paragraphs below describe the changes in laminin, together with relevant inflammatory and BBB markers, present in studies involving stroke with a transient occlusion. Laminin expression and staining were studied in the brains of animals that had either transient blood flow interruption or hypoxia. Results presented here refer to the ipsilateral or affected brain region. No changes in laminin were observed at 4, 8, or 12 h after transient MCAO. This was true even when the animals with transient MCAO were challenged with IL-1β [37]. In addition, no changes were observed in laminin α2 and α4 subunits 12 h after the reduction in blood flow [38]. Nonetheless, at 16 h post transient MCAO, a significant reduction in laminin was found [35], which persisted from 16 h until 7 days after transient MCAO [36]. In the study of McColl and colleagues [37], neuronal laminin (but not vessel laminin) was found to be significantly reduced 24 h after transient MCAO both in the animals which had MCAO and in those which had MCAO plus IL-1β challenge. Steiner and colleagues [38], examined the laminin subunits α2 and α4 at 24 and 48 h after the transient MCAO, finding a significant reduction in the compromised brain hemisphere at these two timepoints. In another study, laminin was found to be significantly reduced 3 days after the transient MCAO [39]. The authors also found that treatment with the water-soluble carbon monoxide-releasing molecule-3 (CORM-3) restored laminin levels to those of sham controls. Furthermore, in a transient bilateral common carotid artery occlusion experimental model, laminin subunit α4 was found to be significantly decreased in CA1 and CA2 regions of the hippocampus [36]. This reduction in laminin was rescued by treating the CD1 mice with the selective serotonin reuptake inhibitor fluoxetine. Boroujerdi and colleagues [33] placed mice in a controlled low-hypoxic chamber for 14 days, and then examined the effects of this chronic exposure 7 days and 14 days post hypoxia in cerebral blood vessels. Compared with normoxic controls, an augment in fragmented laminin-positive cells (which correlates with reduced active laminin) was found at day 14 under hypoxia, as well as at days 7 and 14 post hypoxia. The largest increase was reported at 14 days post hypoxia. In addition, animals, which had the MMP-9 gene knockout, showed a significant reduction in fragmented laminin-positive cells compared with wildtype animals, at day 16 post hypoxia. All these studies showed some type of reduction in laminin, measured from 16 h to 14 days after the experimental procedure. This reduction seems to be based upon decreased expression of α2 and α4 subunits.

Changes in laminin expression were related to changes in other proteins from the ECM or from NVU cells. Between 16 and 48 h after transient MCAO, laminin decrease affected other proteins of the basal lamina such as β1-integrin, β-DG, collagen-IV, and agrin. This decrease in laminin corresponded with an increased expression of matrix metalloproteinases (MMPs), cytokines, and proinflammatory chemokines in the injured regions [35,37]. At 16 h, in an ischemia/reperfusion (I/R) injury model, laminin expression decreased, accompanied by an increase in glial fibrillary acidic protein (GFAP) expression levels and a reduction in pericyte expression marked with alpha smooth muscle actin (α-SMA) [35]. On the other hand, between 24 and 48 h, a decrease in expression of laminin subunits α4 and α2, together with reduction in aquaporin-4 (AQP4) (only at 48 h) and β-DG levels, was found [38]. However, in a more recent study, Kim and colleagues [35] reported a significant increase in AQP4 expression and in the AQP4/GFAP area ratio at 16 h after blood flow interruption. In the same study, treatment with the endothelial dysfunction blocker CU06-1004 restored both markers to sham control levels. Between 3 and 7 days after the transient MCAO, the BBB stability was compromised due to TJ degradation, a decrease in occludin and zonula occludens-1 (ZO-1) protein levels, and an increase in leukocyte infiltration [36,39]. In addition, within this timeframe, the expression of laminin decreased, while an increase in microglial cell proliferation was observed. During this period, GFAP expression was also increased [36], while pericytes, marked with PDGFRβ, were reduced after the transient occlusion [39]. In addition, 14 days after chronic hypoxia, vascular laminin mesh presented a fragmented and disrupted pattern; moreover, expression of claudin-5 in wildtype mouse brains was strongly increased following 14 days hypoxia, whereas, after 14 days post hypoxia, vascular claudin-5 showed a weaker disrupted pattern, and its levels resembled those of normoxic conditions [33]. These results showed that there seems to be a critical time, initiating in particular between 16 and 48 h, and continuing for various days, where several cellular and molecular adjustments occur after an episode of transient ischemia. Concomitant with a decrease in laminin, microglia and astrocytes seem to be more reactive, while pericytes are diminished, and endothelial cells express fewer TJ proteins.

MMP-2 and MMP-9 are considered molecular biomarkers of neuroinflammation in stroke studies. These proteins showed an increased expression and proteolytic activity between 8 h and 7 days, for transient MCAO, and between 7 days and 14 days, for chronic hypoxia [36,37,39]. β-DG, which colocalizes with MMP-9 and serves as an indirect marker of neuroinflammation, was found to show a 32% reduction at 24 h post reperfusion and 11% reduction at 48 h post reperfusion, suggesting increased MMP-9 activity [38]. Similar results were obtained in another study, where β-DG and the β-DG/CD31 area ratio were found to be significantly decreased at 16 h [35]. Accordingly, an increased expression of proinflammatory cytokines (TNF-α, IL-1β, IL-6, COX-2, and iNOS) and chemokines (Gro-α (CXCL-1), MCP-1, MIP-1α, MIP-1β (CCL-4), and MIP-2α (CXCL-2)) was found [36,39]. The increased expression of MMPs and proinflammatory cytokines correlate with the reduction in laminin.

### 3.3. Cerebral Hemorrhage

Intracerebral hemorrhage (ICH) accounts for 10–15% of all strokes and is associated with a high incidence in elderly population [72]. ICH can be caused by aneurisms, hypertension, and arteriovenous malformations, among others. Severe inflammatory processes have been associated with ICH, including hemoglobin degradation, microglial activation, and the liberation of proinflammatory cytokines and chemokines causing peripheral inflammatory infiltration [73]. Laminin is also involved in ICH pathological features, as loss of endothelial laminin α5 is related to elevated BBB permeability and exacerbated hemorrhagic brain injury [24].

Five studies conducted on different cerebral hemorrhage animal models met the inclusion criteria and were incorporated in this systematic review. Three studies used a subarachnoid hemorrhage (SAH) model [41,42,44]. One induced cerebral microhemorrhages (CMH) with LPS (1 mg/kg) [43], and the other induced intracerebral hemorrhage with collagenase type VII-S [40]. Two studies directly injected blood into the pre-chiasmatic cistern [42,44], while the other perforated the bifurcation between anterior and middle cerebral artery [40]. Four of these studies used male Sprague-Dawley rats [41,42,43,44], while one used male and female C57BL/6J mice [40]. Laminin expression was found to be reduced in all the studies associated with animal models of cerebral hemorrhage at every timepoint examined. However, no isoform or specific subunits of laminin were explored in any of the studies.

The paragraphs below describe the changes in laminin, together with relevant inflammatory and BBB markers, present in studies involving cerebral hemorrhage. Two studies explored the outcomes and changes of BBB permeability and laminin expression between 24 and 72 h post SAH [41,44]. It was found that, 24 h after SAH induction, laminin expression was reduced in cortical microvascular walls, accompanied by a reduction in ZO-1 protein [41]. These events triggered a neuroinflammatory response caused by NF-κB activation, followed by an increase in MMP-9, IL-1β, and TNF-α levels, and they also led to BBB extravasation in all brain regions examined, together with neutrophil infiltration and an increase in activated microglia [41,44]. All these changes were accompanied by augmented BBB permeability. At 72 h after SAH induction, occludin and ZO-1 levels were decreased, and laminin expression was also diminished at hippocampal vessels [41]. A model of CMH was established with intraperitoneal (i.p.) injection of LPS at three different timepoints (0, 6, and 24 h) [43]. In this experimental model, it was found that, after 7 days, laminin expression and ZO-1 protein level presented a reduction associated with BBB damage. In addition, the numbers of astrocytes marked with GFAP and of microglia marked with Iba-1 were significantly higher after LPS administration, indicating a neuroinflammatory reaction and glial activation. However, the number of pericytes (marked with PDGFRβ) in animals with CMH was significantly lower compared with controls. A subsequent study injected blood into the pre-chiasmatic cistern to induce SAH in Sprague-Dawley rats [42]. The initial damage after SAH induced several changes such as a rise in BBB permeability and a reduction in the expression of laminin and the TJ proteins occludin and ZO-1, accompanied by increased expression of GFAP and VEGFR-2 in the hippocampus. These effects were significantly attenuated in animals that received the sesquiterpene compound β-caryophyllene.

We also reviewed another study, which induced ICH with injected collagenase type VII-S into the striatum [40]. In this study, an increase in the number of astrocytes was observed at days 2, 5, and 14 after ICH, together with an augment in activated microglia with ameboid morphology (between days 5 and 14 after ICH). Pericyte expression was reduced by day 5, but returned to basal levels at day 14 post ICH. These events were accompanied by an increase in laminin degradation (which affected the expression of caveolin 1), and BBB permeability measured by Evans blue [40]. A reduction in laminin expressed in pericytes was accompanied by neutrophil infiltration (Ly6G, CD11b, and CD3^+^) from days 2 to 14 post ICH, due to the damage in the BBB [40].

All these previous results showed that laminin is decreased in different cerebral hemorrhage animal models and at different timepoints, ranging from 24 h to 7 days. These laminin changes parallel a reduction in endothelial TJ proteins, pericyte function, and increased BBB permeability, with an increase in microglial and astrocytic activation.

### 3.4. Traumatic Brain Injury

Traumatic brain injury (TBI) is a closed head injury which causes different degrees of damage to the brain, including inflammation and neuronal death in the injured areas. However, this focal inflammation can progress to secondary brain injury trough the exacerbation of brain edema and neuronal death, disseminating to other brain areas and causing complications that range from hyperthermia to brain death [74]. Laminin may be important for processes of recovery and neovascularization in TBI, due to its key association with astrocytes, pericytes, and the ECM [75].

Five studies conducted on different traumatic brain injury (TBI) animal models met the inclusion criteria and were incorporated in this review. Two studies used PCI3000 PinPoint Precision equipment to produce a controlled cortical impact [48,49], one used a modified Marmarou force impact model [46], one employed thermal injury, in which rats were submerged horizontally in water at 100 °C for 6 s [47], and one study used a cortical spreading depression model [45]. Three studies used male Sprague-Dawley rats and two used male BALB/c mice.

The three studies of controlled cortical impact used either a PCI3000 PinPoint Precision Cortical Impactor in BALB/c mice [48,49] or a Marmarou force impact in Sprague-Dawley rats [46]. It was found that the levels of MMP-9, occludin, ZO-1, laminin in the vascular wall, collagen IV, and integrin β1 were decreased from 6 to 24 h after the controlled cortical impact [48,49]. Moreover, there was an increased expression of AQP-4 in astrocytes, and the animals presented BBB damage with altered permeability [46,48,49]. Furthermore, the studies which used PCI 3000 PinPoint Precision to produce controlled cortical impact also reported a surge in the p65 subunit of NF-κB, leading to a neuroinflammatory response with increased expression and activity of MMP-9, as well as an increase in the expression of TNF-α, iNOS, hypoxia-inducible factor 1 (HIF-1α), and ICAM-1 [48,49]. In the modified Marmarou force impact model, an enhanced expression of HIF-1α, which had an effect on AQP-4 and MMP-9 levels, was reported [46]. In the thermal injury TBI model, conducted on male Sprague-Dawley rats, a significant decrease in collagen IV, fibronectin, and laminin was found, 7 h after the thermal injury. Moreover, there was an increase in the expression and activity of MMP-9, which was associated with alterations in the BBB permeability, measured as a percentage change in water content and formation of brain edema. Lastly, in the cortical spreading depression model using male Sprague-Dawley rats [45], there was a reduction in endothelial barrier antigen (EBA), ZO-1, and laminin in blood vessels of the affected region at 3 to 24 h after TBI induction. Similarly, an increase in MMP-9 and BBB disruption was found. Laminin expression was found to be reduced in all TBI studies and at all of the timepoints explored (from 3 to 24 h). A neuroinflammatory response was present in the TBI models, together with an increase in MMP-9, which may be responsible for the reduction in laminin. In addition, BBB showed augmented permeability due to a reduction in TJ proteins.

### 3.5. Stroke and Alzheimer’s Disease

Three studies conducted on different cerebral ischemia and AD animal models met the inclusion criteria and were incorporated in the present review. One study induced stroke by permanent MCAO in 3xTg mice [52], and the other two studies caused ischemia with endothelin-1 injected into the right striatum (causing permanent damage) in a model of Aβ toxicity, induced by bilateral injection of Aβ25–35 into the lateral ventricles [50,51]. One study [52] used both female and male wildtype and 3xTg mice, while the other two studies used male Wistar rats [50,51].

In the 3xTg mice model, an increase in the expression of laminin and collagen IV in the neocortex, due to ischemic neuroinflammation and AD induction, was reported. This increase was observed 24 h after the permanent MCAO. Additionally, there was an increase in the number of activated microglia in the ischemic area. In the two experiments with Wistar rats [50,51], there was also an increase in laminin expression and in the number of activated microglia, together with a significant increase in AQP4 and GFAP levels in the stroke model compared with the AD plus stroke model at 24 h and up to 28 days later [50]. Moreover, in the stroke plus AD unified models, there was an increase in MMP-9 expression and astrogliosis due to the effect of ischemic neuroinflammation at 24 h. Furthermore, there was an increased vascular leakage detected with dysferlin and basement membrane laminin-positive vessels [51]. These findings were associated with a decreased expression of SMI71 (also known as endothelial barrier antigen) on microvessels, suggesting TJ damage and increased BBB permeability [50,51]. Laminin expression and BBB permeability were found to be increased in all reviewed studies. However, no isoform or specific subunits of laminin were explored in any of the studies. Neuroinflammatory activity was observed as an increase in microglia together with increased expression of MMP-9.

### 3.6. Alzheimer’s Disease and Aging

AD is a neurodegenerative disorder, which represents the most common cause of dementia worldwide. Most AD cases develop sporadically, associated with risk factors such as aging, the presence of ApoE allele ε4, diabetes, and metabolic syndrome, among others [76]. However, direct genetic causes responsible for early-onset AD are also known, involving mutations in genes such as *APP*, *PSEN1*, and *PSEN2* [77]. The characteristic pathological features of AD include the presence of Aβ plaques, neurofibrillary tangles produced by tau hyperphosphorylation, and persistent neuroinflammatory activity [78]. Laminin may also be involved in AD, as it has been reported that this protein directly interacts with Aβ [79], and alterations in this interaction can lead to neuronal dysfunction [80].

Three studies conducted on different AD or aging animal models met the inclusion criteria and were incorporated in this systematic review. One study used 3 week old, 2 month old, and 18 month old Wistar rats (sex of the animals was not specified), and injected IL-1β or TNF-α to induce neuroinflammation [53]. A second study induced AD with full-length Aβ1–42 or reverse peptide Aβ42–1 injected into the dentate gyrus of male Sprague-Dawley rats for 7 days [54]. Lastly, the last study used male EFAD mice generated by crossing APOE-targeted replacement (APOE-TR) mice with female 5xFAD mice. These EFAD mice were administered PBS or LPS via i.p. injection from 4 to 6 months, in order to induce cerebrovascular dysfunction [55].

Regarding laminin, it was found that its expression was reduced in two of the studies associated with AD [53,55], while one study presented an increase in laminin following 7 days of Aβ1–42 injections in the hippocampus [54]. However, no isoform or specific subunits of laminin were explored in any of the studies. In two of the AD studies, it was found that, between 6 and 24 h, laminin-positive vessels and laminin expression were diminished [53,55]. Moreover, in this time interval, AD models with neuroinflammatory induction presented an increase in several cytokines such as MIP-1, IL-6, IL-5, TNF-α, RANTES, G-CSF, IL-1α, and IL-10, as well as augmented BBB permeability [55]. In the study of Ryu and McLarnon [54], an elevation in TNF-α expression was found to affect the BBB permeability, and this was associated with an increase in laminin from the vascular basement membrane. These results were accompanied by an increase in the number of activated microglia and in the number of reactive astrocytes (examined through GFAP) [54]. On the other hand, in the same paper, researchers found that mice injected with thalidomide (100 mg/kg for 7 days) decreased both the expression of TNF-α and the glial reactivity in the hippocampus of the rats.

Microinjections of IL-1β or TNF-α were applied in 3 week old juvenile and 18 month old aged rats, causing an increase in leucocyte recruitment and BBB breakdown [53]. Importantly, there was an association of age and neuroinflammatory induction with a greater sensibility to develop a neuroinflammatory process in young individuals mediated by the increase in the inflammatory cytokines IL-1β and TNF-α and by neutrophil and macrophage infiltration [53]. BBB stability was disrupted, evidenced by decreased laminin associated with a diminished expression of claudin-1 in the youngest individuals, denoting an age-related effect.

### 3.7. Multiple Sclerosis

MS is a chronic CNS autoimmune disease, characterized by demyelination, neuro-axonal damage, inflammation, and reactive gliosis [81]. The most common experimental animal model for this disease is experimental autoimmune encephalomyelitis (EAE), which is induced by immunization with CNS tissue or myelin peptides, such as MOG, myelin basic protein (MBP), and proteolipid protein (PLP) in complete Freund’s adjuvant (CFA) [82]. Laminin may also play a role in MS, as it has been reported that endothelial laminin facilitates the recruitment of T cells across the BBB under inflammation [83,84].

Two studies conducted in animal models of MS met the inclusion criteria and were incorporated in this systematic review. Both studies induced a model of MS onto 8 to 10 week old female C57BL/6 mice. Wesler’s study also used β4 integrin flox/flox transgenic mice), via immunization with MOG33–35 peptide [56,57]. The study by Welser and colleagues [57] also used CFA containing *Mycobacterium tuberculosis*, and the mice received an i.p. injection of pertussis toxin. The clinical EAE status reached onset at days 12–14 after immunization, with clinical severity signs around 18–21 days (acute phase) and improvement without complete recovery at 32–35 days (chronic phase) [57]. The β4-EC-KO mice showed the same EAE onset time as the wildtype littermates. In the other study [52], NG2 KO and wildtype mice were used.

In the study of Welser et al. [57], the laminin interaction with the laminin receptor integrin α6β4 was examined in order to evaluate their contribution to BBB integrity. β4 integrin expression levels were measured as a CD31/β4 integrin dual immunofluorescence staining on blood vessels. A significantly increased expression of β4 integrin on blood vessels was observed at 7 days post immunization. The number of blood vessels positive for the expression of β4 integrin increased progressively up to the acute stage (day 21) and maintained their augmented levels at day 35 (chronic stage). In β4-EC KO mice, the lack of endothelial β4 integrin resulted in an upregulated expression of neuroinflammatory markers and worsened clinical EAE development [57]. In the same study, an increment in the number of positive cells to MHC II and CD45^+^ inflammatory leukocytes, as well as increased expression of the microglial/macrophage marker Mac-1 associated with leukocyte infiltration in the EAE model, was found. Moreover, the lack of endothelial β4 integrin also impaired the integrity of the BBB, which is represented by more fibrinogen leaky vessels and decreased expression of the TJ proteins, claudin-5, and ZO-1 during the acute stage of EAE.

In the study by Girolamo et al. [56], an increase in laminin was observed in the EAE mice at 20 days post immunization, while the NG2 KO and EAE-affected NG2 KO mice had a markedly reduced expression of collagen IV, collagen VI, and laminin. Moreover, BBB permeability was augmented in EAE mice with a high number of leaky microvessels due to the damage of TJ by the reduced expression of claudin-5 and occludin. The expression of claudin-5 and occludin remained unaffected in wildtype mice. Furthermore, both NG2/CD13 and NG2/PDGFRβ were used as markers for activated pericytes in the perivascular region. In this aspect, in EAE-affected mice, NG2/CD13 pericytes were significantly incremented at 20 days post immunization. Similarly, in naïve NG2 KO and EAE mice, a significant increase in PDGFRβ^+^ pericytes was found compared with wildtype mice.

Lastly, in EAE mice, the expression of several factors from the NVU was measured. For example, VEGF was expressed at the end-feet of perivascular astrocytes measured by immunostaining colocalization with GFAP. Fibroblast growth factor 2 (FGF2) and its receptor FGFR1 were colocalized on the endothelial cells, and PDGF-AA/PDGFRα and PDGF-AA/PDGFRβ were also colocalized on the endothelial cells and the astrocyte-like processes [56].

These results indicated that laminin expression was increased in both studies associated with MS, although no additional information was given regarding specific laminin isoforms or subunits. In addition, β4 integrin, a laminin receptor, also showed increased expression. A lack of this receptor was accompanied by worsened clinical EAE outcome and augmented neuroinflammation. Moreover, the presence of NG2 seems to be important for laminin expression, as knockout animals for this marker showed reduced laminin expression, even under neuroinflammatory conditions.

### 3.8. Status Epilepticus

Epilepsy represents a group of neurological disorders characterized by recurrent seizures that can vary from brief absence episodes to continuous tonic–clonic motor activity. The seizures are a product of abnormal electrical activity of the brain, frequently caused by either excessive excitatory activity (glutamate) or reduced inhibitory control (GABA) [85]. Continued seizure activity may develop into SE, which is associated with both morbidity and mortality. Furthermore, the presence of inflammation has been directly related, as both a cause and a consequence, to epilepsy. For instance, it has been shown that recurrent seizures can induce chronic brain inflammation [86,87]. On the other hand, pre-existing inflammation may increase the propensity to present both seizures and defects in neuronal excitability [88]. Laminin seems to be involved in some aspects of epilepsy and SE, as it has been shown that BBB integrity is compromised in SE when the laminin receptor is dysfunctional [59]. Furthermore, the expression of laminin was reported to be increased in the anterior temporal neocortex tissue from patients with intractable epilepsy [89].

Three studies conducted on different SE animal models met the inclusion criteria and were incorporated in the review. One study induced SE in male 7 week old Sprague-Dawley rats, pretreated with an i.p. injection of LiCl (127 mg/kg i.p.) 24 h before receiving pilocarpine (30 mg/kg) [59]. A second study induced SE in 7 week old male Sprague-Dawley rats pretreated with LiCl (127 mg/kg i.p.) 20 h before receiving pilocarpine (25 mg/kg, i.p.) [58]. Lastly, one study employed kainic acid (10 mg/kg i.p.) and three subcutaneous doses of N3-nitropropionic acid (20 mg/kg) in male Sprague-Dawley rats [60].

In the reviewed studies, laminin expression was significantly upregulated in the vessels recovered from 3 to 4 days post induction of SE in the striatum, hippocampus, and piriform cortex. Laminin expression returned to non-SE levels 1 to 4 weeks after SE [58,59,60]. Furthermore, p38 MAPK and VEGF expression was also increased [59]. Moreover, after KA injection, CD11b-positive microglia activated cells appeared mainly in areas such as the striatum, hippocampus septum, cortex, amygdala, and thalamus, where laminin was also increased [60]. There was an absence of SMI-71 (EBA) labeling, a BBB biomarker, in injured areas of the striatum and hippocampus, or reduced expression in the cortex and thalamus, which evidenced increased BBB permeability [58,59,60].

Astrocytes also presented changes after SE in the hippocampus and piriform cortex [58,59]. In the hippocampus, total GFAP expression increased from 3 days to 4 weeks after SE. CA1 and CA3 hippocampal regions presented increased GFAP expression in most vessels. Four weeks after SE, astrocytes showed typical reactive gliosis in the dentate gyrus. At this timepoint, laminin expression level was similar to that observed in non-SE animals [58]. In the dentate gyrus, 4 days after SE, GFAP expression was reduced in the molecular layer on the basement membrane in astrocytes facing the BBB [54]. Additionally, at this same time, the astroglial 67 kDa LR expression was downregulated in the piriform cortex, which led to a reduction in the expression of the BBB anchor protein dystrophin complex at the astrocyte end-feet. This event was accompanied by a reduction in AQP4 expression and astroglial loss at the basement membrane of the NVU [59].

Laminin expression was found to be increased in all the studies associated with animal models of SE, but no information was given regarding the specificity of the studied isoforms or subunits. Furthermore, laminin returned to basal levels 1 to 4 weeks after the SE, which suggests a recovery in the ECM function. Increased microglia and BBB permeability were observed as a consequence of SE.

### 3.9. Pharmacological and Toxicological Interventions

To induce neurotoxic effects, MDMA, METH, and 3-chloro-1,2-propanediol (3-MCPD) were used in different animal models, leading to neuroinflammatory hallmarks. MDMA is recognized for causing microglial activation, IL-1 changes, and BBB dysfunction [90,91,92]. Likewise, METH induces reactive microglial cell changes and augments the secretion of proinflammatory molecules [93,94], whereas the food contaminant 3-MCPD leads to astrocyte loss, neuronal death, and BBB breakdown [95].

Five studies, conducted on different pharmacological and toxicological interventions, which induced neuroinflammation and barrier breakdown in specific animal models, met the inclusion criteria and were incorporated in this systematic review. The first study used wildtype C57Bl/10 RIII H2 male mice [61], a second study used C57BL/6J male mice [64], a third used male Fisher F344 rats [65], a fourth used Sprague-Dawley rats (sex not specified) [62], and the last one used Dark Agouti male rats [63]. Four of the five studies indicated a decrease in laminin expression during treatments [61,62,63], and only one presented a significant increase in laminin [65].

In a study using male Dark Agouti rats, it was found that the expression of laminin and collagen-IV decreased from 1 h to 6 h after MDMA induction (10 mg/kg) [63]. In addition, these events were associated with increased MMP-3 and MMP-9 activity, IgG leakage at 3 h due to BBB disruption, and an increased number of activated microglia at 6 h. Similar changes were observed in a study using adult male C57BL/6J mice [64]. After 1 h of METH administration in the striatum (three injections of methamphetamine 4 mg/kg, i.p.), reduced laminin expression, increased MMP-9 activity, and BBB leakage as measured by IgG immunostaining were observed [64]. Therefore, MDMA induces an increase in MMP-3 and MMP-9, which may be responsible for the observed reduction in laminin.

In contrast, in a study that used male Fisher F344 rats, after 2 days of 3-MCPD administration (140 mg/kg), laminin immunoreactivity increased and became denser around vascular endothelial cells [65]. GFAP immunoreactive astrocytes were markedly reduced in the inferior colliculus after 2 days, and, at 4 days post treatment, laminin profiles were more irregular around the vascular endothelium. At day 8, laminin profiles were highly inconsistent, and laminin immunoreactivity was also seen in the parenchyma, which was correlated with macrophage and leukocyte infiltration due to BBB disruption. In contrast, fibronectin expression increased 1 day after injection and continued increasing, reaching a peak at day 3. Furthermore, between days 1 and 3, a decrease in occludin and claudin-5 expression was reported. Accordingly, 3-MCPD induces an increase in endothelial laminin expression, together with a compromise of BBB function.

The neurodegenerative disorder glutaric acidemia type I (GA-I) is characterized by striatal and cortical degeneration, BBB disruption, and gliosis [62]. This disease is caused by the absence of the enzyme glutaryl-CoA dehydrogenase (GCDH), resulting in the accumulation of glutaric and 3-hydroxyglutaric acid in the body [96]. A significant decrease in laminin-positive areas associated with small blood vessels was observed 30 days after the injection of glutaric acid (1 μmol/g body weight) in Sprague-Dawley rat pups [62]. This event showed a correlation with the reduction in the number of astrocytes and pericytes in the same area [62]. Moreover, between 14 and 30 days, occludin and ZO-1 expression were unchanged in all conditions analyzed. However, BBB presented an increased permeability, possibly generated by a reduced expression of astrocytes and pericytes of the NVU [62].

In another study, neuroinflammation was established with LPS (obtained from *Escherichia coli* serotype) on C57Bl/10 wildtype and MMP-3 KO mice [61]. LPS-treated wildtype animals showed increased degradation of claudin-5, occludin, and laminin α1. This degradation was reduced in the MMP-3 KO, but was markedly increased, particularly for laminin, when cells from MMP-3 KO animals were treated with MMP-3 in vitro. In addition, the authors reported that MMP-3 facilitated MMP-9 delivery to the brain by neutrophils, thus contributing to LPS-induced BBB disruption. In addition, it was observed that MMP-3 colocalized in parenchymal microglia and pericytes embedded in the basal lamina. Therefore, these results indicated that LPS is involved in laminin degradation, through an MMP-3-dependent mechanism.

### 3.10. Pathogen-Induced Neuroinflammation

Two studies conducted on different neuroinflammation animal models induced by pathogens met the inclusion criteria and were incorporated in the systematic review. The first one induced neuroinflammation through i.p. infection with *Toxoplasma gondii* in female Sprague-Dawley rats [66]. The second study used a gp120 injection in the caudate-putamen of Swiss Webster female mice [67]. Both studies presented a decrease in laminin expression during the reported times of the study.

*T. gondii* infection has been shown to induce an exacerbated inflammatory response in the brain [97]. Infection by *T. gondii* can lead to chronic microvascular brain disorders accompanied by endothelial dysfunction and sustained leukocyte–endothelium interaction, which triggers increased vascular inflammation during the asymptomatic phase of the disease [66]. The induction of neuroinflammation through direct infection of the cerebral parenchyma with the pathogenic microorganism *T. gondii* showed a critical neuroinflammatory response with augmented BBB permeability [66]. In addition, toxoplasmosis infection caused a relevant immunological activation accompanied by an increase in both rolling and adherent leukocytes at the luminal surface of brain venules and microglial activation. Moreover, a significant drop in laminin expression in the capillaries and BBB permeability was found. However, these deleterious effects were only seen up to 40 days post-infection (DPI), in contrast with the human immunodeficiency virus (HIV) model, which started exhibiting changes before 24 h [66].

The second study assessed the neuroinflammatory effect of HIV-1 infection in brain tissue, which was characterized by its sudden onset and increase in activity and expression of MMP-2 and MMP-9. This increase was followed by a significant disruption in the overall integrity of BBB architecture, which was proven by the considerable depletion of laminin and claudin-5 immunoreactivity. Furthermore, the study showed a compromise of BBB permeability through the increase in Evans blue extravasation from 1 to 24 h post infection [67].

### 3.11. Animal Model for Pericytes

Only one study in this category met the inclusion criteria for this systematic review [69]. Pericytes are essential cells for brain microcirculation and BBB maintenance, and they are also known to synthesize and deposit different laminin isoforms into the basement membrane [98]. The included study used PDGFRβ^+/−^ and F7 mutant mice (sex not specified) which significantly reduced the number of pericytes. This animal model showed a potent inflammatory response as recorded through an increase in the levels of the following agents: IL-1β, IL-6, TNF-α, CCL2, and ICAM-1. These alterations impacted the animal’s BBB structure, which presented a decrease in laminin levels and collagen type IV. In addition, BBB permeability was compromised with a reduction in the expression of the TJ proteins claudin-5, occludin and ZO-1, in PDGFRβ^+/−^ mice and F7 mutants. Lastly, in vivo leakage of TMR-dextran was present on all PDGFRβ^+/−^ mice [69].

### 3.12. Postoperative Cognitive Dysfunction

Only one study in this category met the inclusion criteria for this systematic review [68]. This model employed 20 month old male Sprague-Dawley rats that underwent laparotomy surgery to induce postoperative cognitive dysfunction (POCD). The development of POCD is attributed to neuroinflammation during surgery or anesthesia [99]. In the study, at 24 h post surgery, a significant increase in laminin and a decrease in BBB biomarkers such as occludin and ZO-1 was observed, with no changes in the pericyte marker PDGFRβ. This finding was further proven by an increase in S100β serum levels and the expression of heavy chain IgG in the hippocampus [68]. Moreover, the study found an increase in MMP-2 and MMP-9 levels, as well as p-IκBα:IκBα and p-IKKα:β ratios, related to an active neuroinflammatory process. There was also a significant increase in the activity of the inhibitory molecules TIMP-1, TIMP-2, and TIMP-3. However, they did not increase enough to counteract the proteolytic effect of the MMPs. Furthermore, pretreatment with the AT1 antagonist candesartan normalized the expression of laminin.

## 4. Discussion

**Figure 3:**
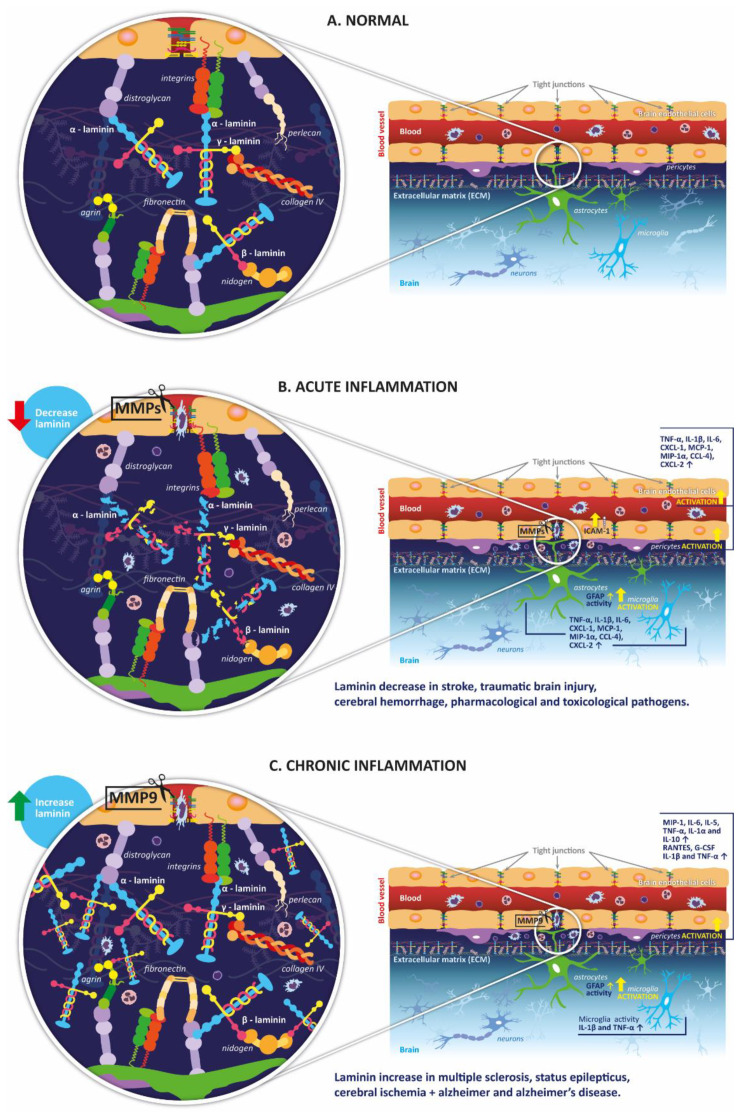
Schematic representation of the main findings in the systematic review regarding laminin. Panel (**A**) represents physiological interactions of laminin in the ECM, and with astrocytes, pericytes, and endothelial cells. Panel (**B**) represents changes in laminin during acute neuroinflammation where a decrease in laminin is reported. Panel (**C**) represents changes in laminin during chronic neuroinflammation where an increase in laminin is reported. Abbreviations: glial fibrillary acidic protein (GFAP); granulocyte colony-stimulating factor (G-CSF); interleukin (IL); macrophage inflammatory proteins (MIP); matrix metalloproteinases (MMP); monocyte chemoattractant protein 1 (MCP-1); tumor necrosis factor (TNF).

In this systematic review, we examined the relationship among neuroinflammation, changes in laminin, and BBB disruption. We found that changes in laminin are related to the specific conditions of the experimental model used in the included studies. Moreover, laminin changes are determined by the presence or absence of permanent damage, as well as by the acute or chronic nature of the induced insult (Figure 3).

Stroke is a useful experimental model to evaluate changes in BBB and laminin due not only to the vascular and ECM damage but also to the strong ischemic and postischemic neuroinflammatory activity this model exhibits [5,100]. In stroke, different components of the ECM such as fibrinogen, fibronectin, collagen IV, and laminin have shown a transient upregulation after MCAO, possibly through the activation of COX-2 and microglia [30]. All stroke studies examined in this systematic review reported a change in BBB, laminin, or neuroinflammatory markers. In particular, laminin changes depended on the type of model used to establish blood perfusion interruption, as permanent occlusion led to an increase in laminin expression, whether transient occlusion or hypoxic condition induced a decrease in laminin. Moreover, changes in laminin may also be related to alterations in specific laminin subunits. The expression of β3 and γ1 subunits seems to be affected by permanent damage while the α subunits, particularly α2 and α4, seem to be affected by transient occlusion. The α subunit is the largest of the three and is involved in interactions with cellular receptors such as integrins and dystroglycans, while β and γ subunits interact with ECM molecules such as collagen and nidogen [101]. The results presented in the systematic review are in agreement with these observations. For example, the study by Ji and Tsirka [30] found an increase in collagen IV, which parallels the increase in β and γ subunits in a permanent occlusion stroke model. In transient MCAO models, a reduction in β1-integrin was found, which could be related to a decrease in the laminin α subunit. As only few studies analyzed the effects of stroke models on the specific laminin subunits, future studies should evaluate this aspect in order to confirm if there is a relationship between transient or permanent damage and laminin subunits. Moreover, further studies are needed to assess whether stroke, together with the accompanying alterations in BBB and neuroinflammation, has any distinctive effects on the different configurations of brain endothelial laminins, such as 411 and 511, or even on less explored cellular laminin configurations such as 323 or 423 [102]. This information regarding subunits and configurations may be crucial for the implementation of laminin as a potential biomarker in stroke models. Data from bioinformatics and cell culture studies may also be of help and offer promising starting points for the development of biomarkers. For example, in a recent study, laminin β1 was found to be the target of peptide-88 under neuroinflammatory conditions in vitro, using the human brain endothelial cell line hCMEC [103].

Time is an essential element in experimental stroke models and in clinical practice, as longer occlusion times represent a greater damage on cells and tissues, together with poor prognosis and higher mortality. In animals which had permanent occlusion, laminin was found to be increased as early as 6 to 12 h, and remained increased up to 7 days after the injury [30,34]. It is still not clear at which precise timepoint laminin starts to increase its expression, as there was no earlier measurement of laminin in the permanent stroke models. However, the longest transient occlusion explored was 90 min before reperfusion [35], which does not seem enough to induce an increase in laminin. Therefore, the increased expression in laminin due to permanent damage seems to develop between 90 min and 6 h. However, the effects on laminin expression produced by reperfusion are still not clear. This may be important, as an increase in laminin may be related to or signal to the presence of permanent damage in the affected brain region. In ischemic stroke, clinical guidelines such as the American Heart Association/American Stroke Association (AHA/ASA) define clear timepoints where emergency interventions (for example, fibrinolysis) can be implemented. In most medical centers, these decisions are based on clinical scales, mainly the National Institutes of Health Stroke Scale (NIHSS), CAT scan or MRI images, or the history of the patient. Although this protocol is very useful and effective in short times after the stroke (mainly 1 h), a gray area for how to proceed challenges clinicians at longer times, especially around 5 h or more. Some groups assume that damage is established at those times or there is a high risk of hemorrhage, precluding fibrinolytic interventions. A more precise biomarker, based on specific pathological changes, could be of extreme utility to clinicians as to determine which patient may benefit from reperfusion, even at longer periods of occlusion.

Stroke, as a model of BBB alterations and neuroinflammation, showed overall consistency in its results. All examined studies showed a significant neuroinflammatory response and subsequent laminin alterations, most of them with reduced expression levels after transient ischemia or hypoxia. This phenomenon may be explained as a result of the physical and functional changes in the brain after an ischemic event [39]. The severe reduction in oxygen delivery to this tissue triggers an immune response and, therefore, activates the neurovascular proteolytic activity of the NVU, mainly through MMP-9 [41]. An increase in MMP-9 expression has been shown to degrade laminin, which even led to neuronal apoptosis, in a transient focal cerebral ischemia model in mice [104]. Consequently, MMP-9 may cause a severe reduction in all basement membrane (BM) proteins, including laminin, leading to a more permeable BBB [33,38]. Further studies should examine specific differences between neuronal-associated laminin (or other brain cells laminin) and the brain endothelial laminin in stroke models.

COX-2 seems to play a role on laminin expression in permanent MCAO. COX-2 is primarily an inducible enzyme that promotes inflammation, although it is also constitutively expressed (in the absence of inflammation) in several organs including the kidneys, gastrointestinal tract, brain, and thymus [105]. In addition, COX-2 is a therapeutic target for nonsteroidal anti-inflammatory drugs (NSAIDs) such as the coxibs. In the study by Ji and Tsirka [30], COX-2 KO animals which had permanent MCAO showed no changes in the expression of laminin α2, β3, and γ1 subunits from 6 to 12 h, as opposed to wildtype animals, which had increased expression of these subunits under permanent MCAO at the same timepoints. This suggests COX-2 may be important for the increase in laminin expression. COX-2 has been shown to be induced during stroke and to enhance inflammatory reactions through the release of enzymatic products, such as prostaglandin E2 [106]. It has also been reported that the effects of COX-2 on laminin expression may be mediated through prostaglandin E2 EP3 receptors [30]. In permanent MCAO models, an increase in laminin expression may be explained by two factors. On the one hand, laminin upregulation has been linked to wound healing stimulation [107]; on the other hand, angiogenesis and neurogenesis are proposed to be coupled by VEGF and laminin/β1-integrin signaling [108]. No included study used COX-2 KO animals in a transient stroke model; therefore, it would be interesting to perform a similar investigation to the one performed by Ji and Tsirka, but with a transient MCAO, as this could be very helpful to clarify the effects of COX-2 signaling on laminin subunits in these two different stroke settings. Furthermore, effects of COX-2 inhibitors and other NSAIDs on brain laminin expression should be investigated in both temporal and permanent occlusion, in order to clarify the effects of these widely used drugs on stroke models. In addition to COX-2 inhibitors, other pharmacological treatments such as fluoxetine have been shown to have regulatory properties on laminin expression. Treatment with fluoxetine rescued the loss of laminin and occludin in the hippocampus after transient bilateral occlusion of common carotid arteries [36]. However, it is not clear if fluoxetine can directly affect laminin expression. Most probably, fluoxetine exerts an indirect effect due to reduction in inflammatory agents or other molecules (such as MMP-2, MMP-9, or COX-2, for example). In contrast, animal knockouts for either CCR2 or CX3CR1 which had permanent MCAO, failed to affect laminin expression, suggesting that these pathways are not involved in laminin structural or functional properties [34].

ICH is a major health problem resulting from the rupture of blood vessels in the brain, and it is related to other factors such as hypertension-related degenerative changes or cerebral amyloid angiopathy [109]. Previous studies have shown that loss of mural cell-derived laminin exacerbates transcellular transport after ICH through a reduction in caveolin-1 and compromises the regulation of brain water homeostasis [40]. In the reviewed models of subarachnoid hemorrhage (SAH), laminin expression was reduced after 24 h, together with an increase in the activation of microglia and astrocytes, accompanied by augmented BBB permeability [42,44]. The increased presence of proteases such as MMP-9 helps to explain the degradation of ECM proteins, including laminin, and the disruption of the BBB due to the loss of TJ proteins [110]. The severity of BBB disruption and the presence of neuronal apoptosis after the injury process may be determined by the balance between MMP-9 and its natural inhibitor TIMP-1 [111]. As the expression of both is regulated by the NF-κB pathway, which is also one of the central intracellular inflammatory pathways, a tight inflammatory control is essential for an adequate BBB and ECM environment [42,44]. In addition, it has been shown that activated microglia, along with infiltrating neutrophils, are critical sources of proinflammatory cytokines and MMP-9 in the brain [112]. Moreover, IL-1β and TNF-α have been reported to be key mediators in MMP-9 production [113]. Administration of recombinant osteopontin (r-OPN) has been shown to prevent early brain injury and BBB disruption in post-SAH, improving the balance between proteolytic (MMP-9) and matrix stabilizing factors (TIMP-1) [41]. The decrease in laminin expression in SAH models was observed at all the timepoints examined (ranging from 24 h to 7 days). It was also correlated with a decrease in occludin and ZO-1 levels, and with the pericyte marker PDGFR-β. As a major component of the BM, laminin plays an essential role in BBB regulation during cerebral hemorrhage. Hence, a decrease in laminin expression exacerbates cerebral hemorrhage, as shown in KO mice models for laminin α5 subunit [24,40]. However, the specific laminin isoforms were not explored in the reviewed SAH articles. This aspect should be examined in future investigations. Some results also indicated a reduction in pericytes, which are important factors for the functional maintenance of BBB. Moreover, previous studies have shown that mutations in either PDGFβ or PDGFRβ diminish pericyte recruitment and result in severe BBB disruption and massive hemorrhage, suggesting the importance of this protein in hemorrhagic processes [114,115].

TBI is an important cause of morbidity and mortality worldwide. The brain damage varies amply depending on the type and intensity of the traumatic force, ranging from localized small injuries to critical diffuse lesions leading to death [116]. As TBI produces brain tissular damage, many pathological mechanisms regarding laminin responses are shared with ICH or ischemia. For example, all the TBI studies included in this systematic review showed a reduction in laminin, and a similar trend was observed in the studies conducted in ICH and transient ischemic stroke models. However, other studies in CCI models have reported an increase in laminin and other proteins such as fibronectin and tenascin-c after the injury [75,117]. This difference in laminin expression can be explained by the time at which the protein was analyzed, as an increase in laminin was found 7 to 14 days after the injury, while a reduction in laminin was observed 3 to 24 h after the injury. These changes in laminin at different timepoints can be an important indicator of the stage of damage or recovery of the brain tissue, thus supporting the consideration of laminin as a biomarker. TBI results in overexpression of MMPs, capable of degrading the major basal lamina proteins at the ECM, including collagen IV, laminin, fibronectin, and integrin β1 [48]. In addition, MMPs contribute to the loss of barrier function by cleaving TJ structural proteins such as claudin-5, occludin, and ZO-1 [63,118], causing an increase in BBB permeability and favoring the appearance of vasogenic brain edema [46,48,119]. Furthermore, MMPs also contribute to increased expression of proinflammatory cytokines in neurons, astrocytes, and leukocytes, adding to the BBB disruption [49]. The early loss of laminin can be explained by this increase in MMPs activity and hints to adaptive responses to the damage, while later increases in laminin may point to a reparative process.

Previous studies have shown that endothelial cell dysfunction is an important risk factor for the deposition of Aβ peptides and the development of AD [52,120]. In fact, a meta-analysis showed that stroke significantly and independently increased risk for AD, and, in turn, AD increased risk for ICH, suggesting that both conditions share similar pathologic aspects [73]. In stroke and AD unified models, an increase in the expression of laminin associated with inflammation and/or AD induction was observed. TJ damage was also present, associated with BBB disruption and increased expression of astrocytic AQP4. Interestingly, AQP4 is highly important for BBB development and integrity and is involved in the clearance of Aβ in AD [121]. Other markers such as microgliosis and reactive astrogliosis were also present. The three articles that used this model analyzed the BBB, laminin, and neuroinflammation results only through immunohistochemistry or immunofluorescence; therefore, more studies using other experimental techniques, such as molecular biology approaches, are needed to support these results. However, the three articles reported an increase in laminin using a model of permanent vascular damage (permanent MCAO or with endothelin-1 injection), obtaining similar results to those mentioned above in the stroke section, where permanent damage also was associated with increased laminin.

Different studies have shown a chronic inflammatory response during AD development, due to the activation of astrocytes, microglia, and the release of numerous cytokines including IL-1β and IL-6, which in turn contribute to increased Aβ production and tau hyperphosphorylation [122,123,124]. Similarly, alterations in laminin expression patterns of α1 and γ1 laminins have been observed in AD human brain tissue [125]. In AD models, a decrease in laminin expression in vessels was found (from 6 to 24 h), together with an increase in proinflammatory cytokines, which compromised BBB function [56]. In AD patients, this inflammation seems to be related to a protective role during the initial phase, but becomes detrimental in later phases of the disease, suggesting an evident decrease in cellular protection mechanisms. [124]. Some studies have shown an elevation in IL-1 levels correlated with increased APP production, Aβ load, and activated microglia [124,126]. Previous studies observed a colocalization between laminin and amyloid plaques on rat primary hippocampal neurons [79], as well as laminin subunit α1 overexpression in the frontal cortex of AD brains [125]. Moreover, an age-related reduction in laminin was observed in the striatum of Wistar rats [53]. It has been reported that aging is related to both increased glial cell reactivity (astrocytes and microglia) and functional alterations in the BBB [127]. This may explain why a difference in laminin expression was found between juvenile and aged rats. The two AD models produced opposing results regarding laminin, as the study conducted by Ryu and McLarnon [54] showed an increase in laminin, while the study by Marottoli and colleagues [55] found a decrease in laminin. Several factors may explain this contrast, such as a different experimental model (Aβ peptide injection vs. LPS-injection in AD transgenic animals), different species (Sprague-Dawley rats vs. transgenic mice), or different treatment times (Aβ treatment for 7 days vs. LPS treatment for a couple of months). Overall, these outcomes highlight the relevance of changes in laminin expression for the pathogenesis of the disease, and suggest the importance of further studies examining the integrity of BBB under neuroinflammation in AD and in aging.

EAE is the most commonly used experimental model for the study of MS [22,56,57]. Previous studies have shown that, in the brains of mice with EAE, inflammatory perivascular cuffs and leukocyte infiltration zones are associated with laminin α4 and α5, present in the endothelial BM [83,128]. In this systematic review, we found that, during the acute EAE phase, the increased expression of laminin is produced in parallel with altered integrity of the BBB, represented by a loss of TJ proteins [56]. As previously mentioned, laminin is one of the most abundant components of the BM and plays a vital role in the differentiation and stabilization of vascular endothelial cells [129,130,131,132,133,134]. The components of the BM can modulate the activation and infiltration of leukocytes in the CNS, resulting in inflammatory responses. Furthermore, leukocytes express specific laminin receptors, which regulate their migration and infiltration through the BBB [135]. Thus, in the EAE model, the massive infiltration of leukocytes is facilitated by the type of laminin overexpressed in the BM of the vascular endothelium [57]. In this aspect, previous studies have shown that lymphocyte migration and extravasation through the vascular endothelium correlate preferentially with the interactions between α6β1 integrin from lymphocytes and overexpressed laminin-411 (α4, β1, and γ1 chains), as well as low expression of laminin-511 (α5, β1, and γ1) [83]. Conversely, a higher expression of laminin-511 inhibits T lymphocyte migration, avoiding the interactions between lymphocytes expressing α6β1 integrin and laminin-411 [136]. Moreover, it was found that α6β1 integrin laminin receptors in the normal brain are expressed principally in arterioles; however, once neuroinflammation is established, α6β1 integrins are strongly overexpressed in other brain vessels [14,137,138].

In MS, leukocytes infiltrating into the cerebral parenchyma must first overcome the endothelial barrier and next cross the molecular layer of the endothelial BM, which allows them to reach the perivascular space. Finally, they must overcome the physical barrier given by the glial BM at the astrocyte’s end-feet [139,140,141,142,143]. Once in the perivascular space, leukocytes accumulate until a chemotactic signal induces their transmigration through the glial BM to invade the brain parenchyma. Therefore, the interactions between laminin of the vascular endothelial cells and integrin receptors around astrocytic end-feet are essential to maintain BBB integrity. Thus, the loss of the astrocytic laminin receptor, integrin β4, results in BBB leakage, facilitating the infiltration of immune cells and establishing a neuroinflammatory process [57]. Moreover, this vascular leakage is correlated with a reduced expression of TJ proteins such as claudin-5 and ZO-1 [57]. In EAE, the cells that build the NVU increase the expression of several factors and signaling mechanisms, which affect the endothelial cell functions that regulate BBB integrity, such as PDGF-AA, TGFβ, FGF2, and VEGF-A [56]. For instance, VEGF-A is known to be essential for vessel growth and angiogenesis under normal conditions, and is augmented in EAE and MS in reactive astrocytes [144]. Similarly, FGF2/FGFR1 is also increased in the endothelial cells, which consequently induces VEGF expression [145,146,147]. Furthermore, at the perivascular space, pericyte cells which share the basal lamina with endothelial cells secrete TGF-β, promoting BBB formation [148]. In EAE, TGF-β expressed on the endothelial–pericyte layer could contribute to the survival of endothelial cells [148,149].

SE is a life-threatening condition, which causes significant morbidity and mortality. SE develops when a seizure episode fails to spontaneously resolve, persistently overactivating brain cellular networks, and leading to neuronal death due to oxidative stress, excitotoxicity, and inflammation [150,151]. In addition, SE has been observed to compromise BBB function [152]. For this reason, the role of laminin in epilepsy and in SE has been studied. An early study by Wu et al. (2009) [153] found increased laminin serum level in patients with intractable epilepsy compared with controls, probably as a consequence of reactive gliosis and glial scar formation. Furthermore, a blockade of laminin receptor in SE has been associated with AQP4 downregulation in astrocytes and edema formation [154]. In our systematic review, we found that an increase in laminin expression was observed in all the studies and in different brain areas such as the striatum, piriform cortex, and hippocampus, following the induction of SE. Neuronal damage could promote increased permeability of the vascular endothelium and disruption of the BBB, triggered by alterations in the communication between astrocytes and vascular endothelium. Laminin, as a structural component of the BM, contributes to the preservation of the physical barrier of the gliovascular layer formed by the feet of astrocytes and a mature BBB [133]. After an SE, the expression of the astroglial 67 kDa laminin receptor and the dystrophin–glycoprotein complex decreases. These proteins serve to keep the end-feet of astrocytes anchored to the BM of the BBB. Furthermore, the laminin receptor appears to be related to the astrocytes surrounding the vascular endothelium and to the polarization in AQP4 expression. Afterward, an increase in the astrocyte separation from the BM develops, leading to a disruption in the integrity of the barrier by dissociation of the laminin protein [155,156,157]. At this point, astrocytes can secrete angiogenic factors and induce an increase in VEGF expression, via activation of the p38 MAPK signaling pathway in endothelial cells, leading to a neovascularization process, which increases the permeability of the BBB and decreases the expression of TJ. This results in laminin overexpression, reflecting its crucial role in the organization and proliferation of endothelial cells during angiogenesis [60].

An increased permeability of the BBB in SE facilitates the leakage of albumin from the blood into the brain tissue, stimulating neuroinflammation due to excessive activation of microglia and astrocytes [50,158,159]. The increase in laminin labeling on damaged blood vessels is due to the degradation of the basal lamina proteins, which may cause laminin epitopes to be more exposed. This implies that the overexpression of laminin can also be secondary to an extravasation process. This may occur in vasogenic edema, where albumin extravasation also reinforces the p38 MAPK/VEGF signaling pathway by decreasing the expression of the laminin receptor, with a compensatory increase in laminin. Thus, it seems that the increase in laminin expression, associated with changes in the permeability of the vascular endothelium, occurs before the onset of reactive astrogliosis. For instance, the development of vasogenic edema after an SE result in increased laminin expression as a compensatory mechanism [58]. This finding promotes the activation and migration of astrocytes into the damaged vessels to help restore the barrier. Lastly, it should be noted that the increase in laminin labeling could be correlated with damage to the BBB and subsequent reactivation of astrocytes after an SE [160].

This systematic review included five studies with different pharmacological and toxicological interventions that induced neuroinflammation [61,62,63,64,65]. Previous studies with drugs that induce alteration of the BBB have found an increase in neuroinflammation and significant changes in ECM proteins, including ZO-1, claudin-5, and laminin. MMPs seem to be crucial, as they form a final common pathway for the neuroinflammatory response in many neurological disorders, degrading components of ECM [157,161]. Indeed, laminin has been identified as a substrate for several MMPs [100,158]. MMP-9 can degenerate several matrix proteins in the cerebrovascular basal lamina, including laminin, collagen IV, and fibronectin. Likewise, MMP-3 contributes to BBB disruption by attacking the basal lamina components during neuroinflammation, degrading various collagens, elastin, fibronectin, and laminin, and activating latent forms of other MMPs such as MMP-9 [63,162].

The effects on laminin of several pharmacological and toxicological agents were examined in our systematic review. In one of the included studies, it was reported that a single dose of MDMA reduced the expression of collagen IV and laminin, together with increased activity of MMP-3 and MMP-9 [63]. Although relatively little is known about the specific role of each laminin isoform after MDMA administration, the laminin α3 subunit has been associated with brain changes due to MDMA-induced serotonin depletion [163]. The activity of both MMPs is related to augmented degradation of the BM proteins, collagen IV, and laminin, which is accompanied by a temporary increment in BBB permeability [63,104,164]. These findings concur with other studies displaying elevated MMP-9 activity after METH administration in the striatum, which was also associated with laminin degradation and increased BBB permeability [64,165]. In addition, a single injection of methamphetamine was enough to induced BBB failure, evidenced by increased Evans blue leakage and strong albumin immunoreactivity in the brains of methamphetamine-treated rats [166]. Furthermore, MDMA can promote the release of proinflammatory cytokines, such as IL-1β, which stimulate MMP-9 activity and may further degrade laminin [36,167,168,169]. In addition, MMP degradation of laminin appears to be regulated by various subtypes of nucleotides (P2X and P2Y) and adenosine (A1, A2A, and A3), which can control the production of inflammatory mediators and cell survival [63,170]. For example, P2X7R is found in various brain cell populations, including microglia [171,172]. MDMA causes overexpression of P2X7R before microglial activation, suggesting that enhanced P2X7R expression may trigger microglial activation and proliferation [170], favoring their transition to a reactive state [63]. Therefore, MDMA seems to promote the development of neuroinflammation through IL-1β secretion, MMP activity, laminin degradation, BBB breakdown, and microglial activation. Comparably, METH induces similar effects with high MMP-9 activity, reduced laminin expression, and BBB dysfunction.

LPS has also been used as a model of neuroinflammation based on different protocols, by single or multiple injections [173]. It is known that LPS increases the activity of MMPs and stimulates Toll-like receptors (TLRs) on microglia, promoting cytokine expression [174]. As aforementioned, MMPs contribute to neuroinflammation, laminin degradation, and BBB disruption. However, MMP-3 also contributes to LPS-induced disruption of the BBB by facilitating the delivery of MMP-9 to the brain in neutrophils [61]. This finding suggests that both endogenous MMP-9 production and MMP-9 neutrophil delivery occur in the LPS-injected brain. Furthermore, the previous authors reported that pericytes embedded in the basal lamina and parenchymal microglia are positively labeled with MMP-3, suggesting that both cells contribute to the degradation of laminin from the basal lamina and the TJ proteins claudin-5 and occludin, involving MMP-9. Consequently, the loss of these elements facilitates the access of neutrophils into the brain. Notably, the presence of MMP-3 in pericytes is crucial since its release to the basal lamina brings it into contact with laminin, triggering its degradation and exposing TJ proteins to MMP-9 and other proteases or may even be sufficient to breach the BBB [61,104,164].

After administration of the food contaminant 3-chloropropanediol, a progressive increase in laminin immunoreactivity around endothelial vessels, accompanied by rapid loss of GFAP immunoreactivity in astrocytes, was observed [65,175]. The increased expression of laminin and fibronectin was observed when occludin and claudin-5 were absent from the paracellular domains and the vascular leakage of macromolecules ceased. These facts indicate that the ECM is essential in forming a temporary selective barrier. Notably, laminin shows an increase in immunoreactivity that subsequently decreases as the gliovascular connections are established [65]. Thus, the BM is a dynamic element with an outstanding ability to remodel and restore BBB permeability under different challenges [65]. The endothelial BM of the BBB is a rich area of laminin isoforms where each one could be playing a determining role in the BBB remodeling [142,176]; nevertheless, this aspect remains poorly understood. Furthermore, after the loss of GFAP-immunoreactive astrocytes, there was a decrease in TJ proteins and an augment in the permeability of BBB with CD169 peripheral immunoreactive macrophages infiltrating the injured area. Primarily, these macrophages are located at perivascular sites where they can actively contribute to the degradation and remodeling of the ECM by sequestering macromolecules, which escape through an expanded ECM [177]. Therefore, it has been suggested that macrophages infiltrated into the lesion induced by 3-chloropropanediol exhibit an important role in limiting the amount of macromolecular extravasation of the compromised BBB after damage, attempting to restore its integrity [65].

Newborn rats that received an intracisternal administration of GA, developed striatal BBB leakage, NVU alterations, and reduction in the laminin associated with blood vessels [62]. Increased BBB permeability in the striatum, due to GA injection, has been observed in rats at 14 and 30 DPI, a critical period, as it is during this time that the BBB is developed [178,179]. Furthermore, intracisternal injection of GA also caused a reduction in AQP4 and PDGFRβ, suggesting an indirect effect of GA on pericytes [62]. Interestingly, pericyte malfunction has been associated with a dysfunctional BBB [180,181]. The decreased AQP4 immunoreactivity after GA injection indicates structural and possibly functional defects in the astrocytic end-feet, associated with the microvasculature [62]. Notably, the polarized distribution of AQP4 in astrocytes end-feet appears to be mediated by the interaction with perivascular laminin, highlighting its role as a clustering inductor of AQP4 and dystroglycan [182]. However, GA failed to disrupt occludin and ZO-1, suggesting that an interruption of the BBB would not necessarily require decomposition of the endothelial TJ. Other explanations may include increased transendothelial vesicle trafficking or ECM protein alterations, including laminin [62].

Infection with the intracellular parasite *T. gondii* has been recognized as an inductor of an impaired inflammatory response in the brain [97]. Following *T. gondii* inoculation, an interaction between leukocytes and brain endothelial cells is evidenced [66]. This interaction induces the activation of endothelial cells, increasing the expression of membrane adhesion molecules and favoring the recruitment of T cells into the brain [183,184]. Moreover, the activation of microglia and increased levels of the proinflammatory cytokines IL-1β, IL-6, and TNF are hallmarks of chronic *T. gondii* infection in the cerebral cortex and hippocampus [185]. Therefore, the augmented proinflammatory environment present during toxoplasmosis may compromise microglial and endothelial function. This can be supported by experimental evidence, which has shown a reduction in laminin and small vessels, together with altered angiogenesis, in the brains of infected animals [66]. Angiogenesis is critical for vascular remodeling, and abnormalities in this process are found in many pathological events, including migration of endothelial cells and BM remodeling by MMPs [186]. In fact, the cortical capillaries represent the primary access route of the parasite from the bloodstream into the brain, with a preferential tropism for capillaries with a low α5 laminin/P-glycoprotein expression ratio [187]. Altogether, these findings indicate a role of laminin isoforms in the blood vessel stability and the parasite’s entry into the brain. Although only one study that used *T. gondii* infection as a model of neuroinflammation was included, it seems the presence of this parasite in the brain may decrease laminin. More studies are needed to confirm the relationship between *T. gondii* and laminin in the brain and the influence on BBB function due to this infection.

The BBB breakdown can facilitate the entry of several pathogens, including HIV-1, into the CNS. The injection of the HIV-1 envelope glycoprotein Gp120 in the caudate-putamen of rats increased MMP-2 and MMP-9 levels and their gelatinolytic activity, leading to sustained vascular permeability [67]. Gp120 administration also induces a decrease in laminin and claudin-5 in the vascular endothelium, suggesting that MMP activation may lead to laminin and TJ protein degradation and, therefore, BBB disruption. In addition, the reduction in claudin-5 and other TJ proteins has been related to increased transmigration through the brain endothelium of monocytes, dendritic cells, and HIV-infected human leukocytes [188,189,190]. Other changes, such as the induction of ROS in brain endothelial cells by HIV-1 proteins, Gp120 and Tat, have also been observed in previous studies [191,192]. Therefore, Gp120 induces BBB impairment via brain microvessel lesions through an increase in MMPs and oxidative stress, which leads to a reduction in TJ proteins and laminin. In addition, soluble Gp120 impairs astrocytic and neuronal reuptake of glutamate, promoting excitotoxicity and cell death [67,193].

Our systematic review included one study that examined the critical role pericytes play in maintaining BBB barrier integrity during aging. In that study, it was found that deficient PDGFRβ signaling resulted in pericyte degeneration in the brain, leading to BBB breakdown and diminished brain microcirculation [69]. This study reported that a 40% loss in pericyte coverage, in 6–8 month old PDGFRβ^+/−^ mice resulted in severe neurovascular compromise, with reduced expression of TJ and BM proteins, including laminin. Laminin is of paramount importance for BBB, as mice knockout for laminin exhibited BBB disruption and increased permeability, similar to what occurs in increased laminin degradation [40]. Furthermore, the presence of neuroinflammation adds to the BBB disruption, as pericyte-deficient mice display a significant increase in the number of activated microglia [69]. These cells may exert opposite effects on BBB. On the one hand, vessel-associated microglia maintain BBB integrity through expression of claudin-5 and establish physical contact with endothelial cells. On the other hand, microglia during sustained inflammation phagocytose astrocytic end-feet and impair BBB function [194]. However, additional studies will be needed to establish the precise functions of laminin in pericyte maintenance of BBB and the functional or pathologic relations between pericytes and immune cells such as microglia.

In animal models of POCD, the surgical intervention seems to affect the MMP/TIMP balance in the hippocampus, suggesting BBB dysfunction due to surgical trauma [68,195,196]. In the rat model of POCD, an increase in MMP-2 and MMP-9 was reported, most likely caused by the upregulation of angiotensin II [197,198]. The endogenous inhibitors, TIMPs and α-2-macroglobulin, tightly control MMP activity [198]. Generally, all TIMPs can inhibit all known MMPs; however, the efficacy of MMP inhibition varies depending on the type of TIMPs, specific tissue, and local tissue environment [199,200]. The MMP/TIMP imbalance at the translational level detected as early as 6 h after surgery corresponded with the decreased TJ protein expression of occludin and ZO-1 24 h post surgery and increased IgG extravasation into the brain parenchyma. Therefore, the MMP/TIMP balance can be crucial to maintain the expression of TJ proteins [68]. Interestingly, 24 h after surgery, there was a significant increase in laminin, which can be accounted as a compensatory response of the BBB, as proven by the leakage of serum protein IgG from loosened TJs between endothelial cells [65]. Furthermore, it has been reported that cytokines, including TNF, TGF-β, and IL-1β, can upregulate laminins α4 and α5 in tissues with inflammation [201].

The BBB integrity depends on numerous cellular and molecular factors, particularly astrocytes, brain endothelial cells, and pericytes. Laminin is a critical component for the stability of both ECM and BBB. Therefore, alterations in laminin expression or function can have a direct impact on the BBB. The results presented in this systematic review show changes in laminin due to various pathological models, which have BBB alterations and neuroinflammation in common. Specific variations in laminin, including changes in the three main subunits or in the different configurations, can be explored in diverse pathologies known to compromise the BBB. For instance, changes in laminin (or one of its subunits) at different timepoints may indicate if a pathological process is in a damaging or recovery phase, which may be of use for clinicians, particularly for diseases such as stroke or MS. In addition, laminin may also serve as an indicator of therapeutic success, as it may represent the functionality of the BBB after surgical or pharmacological treatment. Thus, we suggest laminin as a possible biomarker candidate for BBB changes in a diverse range of pathological conditions.

## 5. Conclusions

There is limited knowledge about the role that the different isoforms of laminin play in maintaining the structural architecture of the BBB or its function as an immune cell mediator. However, certain studies highlighted the role of laminin-511 (α5-β1-γ1) and laminin-411 (α4-β1-γ1), which are differentially expressed in BBB under inflammatory conditions. In this aspect, a higher expression of laminin-511 has been reported to improve the BBB integrity by stabilizing the TJ and reducing leukocyte extravasation under neuroinflammatory conditions.

When neuroinflammation is quickly established, the decrease in laminin expression is associated with a disruption of the BBB due to the degradation of TJ induced by the immunological activation of astrocytes, pericytes and microglia. However, in the case of a progressive neuroinflammatory process, laminin expression is increased over a time course. Likewise, in ischemic stroke, permanent vascular damage seems to correlate with increased laminin expression, while transient damage seems to correlate with reduced laminin expression. In addition, laminin was decreased in all the animal models of cerebral hemorrhage and in TBI.

In different disease models it was observed that the communication between astrocytes and vascular endothelial cells could be altered, leading to an increased BBB permeability, which in turn is associated with an increased laminin expression. Once astrocytes and pericytes detect these changes in laminin expression, they can promote immune cell infiltration and BBB damage in a context of neuroinflammatory progression, which also induces a decrease in laminin expression and BBB disruption. Laminin seems to be closely related to the regulation and polarization of astrocytes, as they act as anchors for astrocytic end-feet and promote the expression of AQP4. Is not clear if these changes in laminin affect the function of the glymphatic system. Furthermore, laminin disruption may be an important precursor of the reactive gliosis seen in many pathological processes. These immunological changes, in turn, produce an increase in MMPs that will trigger a decrease in the expression of laminin, leading to TJ damage and BBB disruption. This can be evidenced both in a direct induction of neuroinflammation and in disease models.

The reviewed studies showed that laminin may be a good indicator of the overall structural integrity of the BBB under neuroinflammation. This suggests that laminin expression could serve as a possible biomarker of BBB alterations.

## Figures and Tables

**Table 2:** Risk of bias assessment.

Reference	Allocation Sequence Adequately Generated and Applied? (Selection)	Were the Groups Similar at Baseline? (Selection)	Was the Allocation Adequately Concealed? (Selection)	Were the Animals Randomly Housed during the Experiment? (Performance)	Were the Caregivers and/or Investigators Blinded to the Knowledge? (Performance)	Were Animals Selected at Random for the Outcome Assessment? (Detection)	Was the Outcome Assessor Blinded? (Detection)	Were Incomplete Outcome Data Adequately Addressed? (Attrition)	Are Reports of the Study Free of Selective Outcome Reporting? (Reporting)	Was the Study Apparently Free of Other Problems that Could Pose a High Risk of Bias? (Other)
McColl BW et al. (2008) [37]	N/A	N/A	N/A	N/A	Yes	N/A	Yes	N/A	N/A	Yes
Ji K, Tsirka SE. (2012) [30]	N/A	N/A	N/A	N/A	N/A	N/A	N/A	N/A	Yes	Yes
Steiner E et al. (2012) [38]	N/A	N/A	N/A	N/A	N/A	N/A	N/A	Yes	Yes	Yes
Lee JY et al. (2014) [36]	N/A	N/A	N/A	N/A	N/A	N/A	Yes	No	Yes	Yes
Boroujerdi A et al. (2015) [33]	N/A	N/A	N/A	Yes	N/A	N/A	N/A	N/A	Yes	Yes
Cisbani G et al. (2018) [34]	N/A	N/A	N/A	N/A	N/A	N/A	Yes	Yes	Yes	Yes
Wang J et al. (2018) [39]	Yes	Yes	Yes	Yes	Yes	Yes	Yes	No	Yes	Yes
Kim DY et al. (2020) [35]	N/A	N/A	N/A	N/A	N/A	N/A	N/A	Yes	Yes	Yes
Suzuki H et al. (2010) [41]	N/A	Yes	Yes	N/A	N/A	N/A	Yes	N/A	Yes	Yes
Zhang XS et al. (2015) [44]	N/A	N/A	Yes	Yes	N/A	Yes	Yes	Yes	Yes	No
Zeng J et al. (2018) [43]	N/A	Yes	N/A	N/A	N/A	N/A	N/A	Yes	Yes	No
Gautam J et al. (2020) [40]	N/A	N/A	N/A	N/A	N/A	N/A	Yes	N/A	Yes	Yes
Wang F et al. (2020) [42]	Yes	Yes	Yes	N/A	N/A	N/A	Yes	No	Yes	No
Gursoy-Ozdemir Y et al. (2004) [45]	N/A	N/A	N/A	N/A	N/A	No	N/A	Yes	Yes	Yes
Reyes R et al. (2009) [47]	N/A	Yes	N/A	N/A	N/A	N/A	N/A	N/A	N/A	No
Higashida T et al. (2011) [46]	N/A	Yes	N/A	N/A	N/A	N/A	N/A	Yes	Yes	Yes
Tao X et al. (2015) [48]	Yes	Yes	Yes	N/A	Yes	N/A	N/A	No	No	Yes
Tao XG et al. (2017) [49]	N/A	Yes	Yes	N/A	Yes	Yes	Yes	Yes	Yes	No
Hawkes CA et al. (2013) [52]	N/A	Yes	N/A	Yes	N/A	N/A	N/A	Yes	Yes	Yes
Amtul Z et al. (2018) [50]	N/A	Yes	N/A	N/A	N/A	N/A	N/A	N/A	Yes	Yes
Amtul Z et al. (2019) [51]	N/A	Yes	N/A	N/A	N/A	N/A	Yes	N/A	Yes	Yes
Campbell SJ et al. (2007) [53]	N/A	N/A	N/A	N/A	N/A	N/A	Yes	Yes	Yes	Yes
Ryu JK, McLarnon JG (2008) [54]	N/A	Yes	N/A	N/A	Yes	Yes	N/A	Yes	Yes	Yes
Marottoli FM et al. (2017) [55]	Yes	Yes	Yes	N/A	Yes	Yes	N/A	Yes	Yes	Yes
Welser JV et al. (2017) [57]	N/A	N/A	N/A	N/A	N/A	N/A	N/A	No	No	No
Girolamo F et al. (2019) [56]	N/A	No	No	No	No	N/A	No	No	Yes	Yes
Sarkar S, Schmued L. (2010) [60]	N/A	N/A	N/A	N/A	N/A	N/A	N/A	Yes	Yes	Yes
Kim Y et al. (2014) [58]	N/A	Yes	N/A	N/A	N/A	N/A	Yes	Yes	Yes	Yes
Park H et al. (2019) [59]	N/A	N/A	N/A	N/A	N/A	N/A	N/A	Yes	Yes	Yes
Gurney KJ et al. (2006) [61]	N/A	Yes	N/A	N/A	No	N/A	N/A	Yes	Yes	Yes
Urrutia A et al. (2013) [64]	N/A	N/A	N/A	Yes	N/A	N/A	N/A	No	Yes	Yes
Willis CL et al. (2013) [65]	N/A	N/A	N/A	N/A	N/A	N/A	N/A	No	Yes	Yes
Isasi E et al. (2014) [62]	N/A	N/A	N/A	N/A	N/A	N/A	N/A	No	Yes	Yes
Rubio-Araiz A et al. (2014) [63]	N/A	N/A	N/A	N/A	N/A	N/A	N/A	No	Yes	Yes
Louboutin JP et al. (2010) [67]	N/A	Yes	N/A	Yes	N/A	N/A	N/A	Yes	Yes	Yes
Estato V et al. (2018) [66]	N/A	Yes	No	N/A	No	No	N/A	No	Yes	Yes
Bell RD et al. (2010) [69]	N/A	N/A	N/A	N/A	N/A	N/A	Yes	N/A	No	Yes
Li Z et al. (2016) [68]	N/A	Yes	N/A	N/A	N/A	Yes	Yes	Yes	Yes	Yes

This table presents the individual analysis of each paper using the RoB assessment tool developed by SYRCLE. Biases are grouped into selection, performance, detection, attrition, reporting, and other biases. The responses were “yes” for low risk of bias, “no” for high RoB, and “not available (N/A)” for unclear or unreported risk.

**Table 3:** Study quality assessment.

Reference	Was it Stated That the Experiment Was Randomized at Any Level?	Was it Stated That the Experiment Was Blinded at Any Level?	Publication in Peer-Reviewed Journal?	Statement of Control of Temperature?	Randomization to Treatment or Control?	Blinded Induction of Treatment?	Study Quality—Blinded Assessment of Outcome?	Sample Size Calculation?	Statement of Compliance with Regulatory or Ethical Requirements?	Statement Regarding Possible Conflicts of Interest?	Is There Enough Statistical Power? Is the *n* Number Clearly Reported to Do Statistics Adequately?	The Study Clearly Describes Statistical Analyses?
McColl BW et al. (2008) [37]	No	Yes	Yes	No	No	No	No	No	Yes	No	No	Yes
Ji K, Tsirka SE. (2012) [30]	No	No	Yes	No	No	No	No	No	Yes	Yes	No	Yes
Steiner E et al. (2012) [38]	No	No	Yes	No	No	No	No	No	Yes	Yes	No	Yes
Lee JY et al. (2014) [36]	No	Yes	Yes	Yes	No	No	Yes	No	Yes	No	No	Yes
Boroujerdi A et al. (2015) [33]	No	No	Yes	Yes	No	No	No	No	Yes	Yes	No	Yes
Cisbani G et al. (2018) [34]	No	Yes	Yes	No	No	No	Yes	Yes	Yes	Yes	Yes	Yes
Wang J et al. (2018) [39]	Yes	Yes	Yes	Yes	No	Yes	Yes	No	Yes	No	Yes	Yes
Kim DY et al. (2020) [35]	No	No	Yes	Yes	No	No	No	Yes	Yes	Yes	No	Yes
Suzuki H et al. (2010) [41]	Yes	Yes	Yes	No	Yes	No	Yes	Yes	Yes	Yes	Yes	Yes
Zhang XS et al. (2015) [44]	Yes	Yes	Yes	Yes	Yes	No	Yes	No	No	Yes	Yes	Yes
Zeng J et al. (2018) [43]	No	No	Yes	Yes	No	No	No	No	Yes	Yes	Yes	Yes
Gautam J et al. (2020) [40]	Yes	Yes	Yes	No	No	No	Yes	Yes	Yes	Yes	No	Yes
Wang F et al. (2020) [42]	Yes	Yes	Yes	Yes	No	No	Yes	Yes	Yes	Yes	Yes	Yes
Gursoy-Ozdemir Y et al. (2004) [45]	No	No	Yes	Yes	No	No	No	Yes	Yes	Yes	No	Yes
Reyes R et al. (2009) [47]	No	No	Yes	Yes	No	No	No	No	Yes	Yes	Yes	Yes
Higashida T et al. (2011) [46]	No	No	Yes	No	No	No	No	Yes	Yes	Yes	Yes	Yes
Tao X et al. (2015) [48]	Yes	No	Yes	Yes	Yes	Yes	No	No	Yes	No	Yes	Yes
Tao XG et al. (2017) [49]	Yes	Yes	Yes	Yes	Yes	No	Yes	No	Yes	Yes	Yes	Yes
Hawkes CA et al. (2013) [52]	No	No	Yes	Yes	No	No	No	Yes	Yes	Yes	Yes	Yes
Amtul Z et al. (2018) [50]	No	No	Yes	Yes	No	No	No	Yes	Yes	Yes	Yes	Yes
Amtul Z et al. (2019) [51]	No	Yes	Yes	Yes	No	No	No	Yes	Yes	Yes	Yes	Yes
Campbell SJ et al. (2007) [53]	No	Yes	Yes	Yes	No	No	Yes	No	Yes	No	Yes	Yes
Ryu JK, McLarnon JG (2008) [54]	No	Yes	Yes	No	No	Yes	Yes	Yes	Yes	No	Yes	Yes
Marottoli FM et al. (2017) [55]	Yes	Yes	Yes	No	Yes	Yes	No	Yes	Yes	Yes	Yes	Yes
Welser JV et al. (2017) [57]	No	No	Yes	No	No	No	No	No	Yes	Yes	No	Yes
Girolamo F et al. (2019) [56]	No	No	Yes	No	No	No	No	Yes	Yes	Yes	No	Yes
Sarkar S, Schmued L. (2010) [60]	No	No	Yes	Yes	No	No	No	Yes	Yes	Yes	No	No
Kim Y et al. (2014) [58]	Yes	Yes	Yes	Yes	No	No	No	Yes	Yes	Yes	Yes	Yes
Park H et al. (2019) [59]	Yes	No	Yes	No	No	No	No	Yes	Yes	Yes	No	Yes
Gurney KJ et al. (2006) [61]	No	No	Yes	Yes	No	No	No	Yes	Yes	Yes	Yes	Yes
Urrutia A et al. (2013) [64]	No	No	Yes	Yes	No	No	No	No	Yes	Yes	No	Yes
Willis CL et al. (2013) [65]	No	No	Yes	No	No	No	No	No	Yes	Yes	No	Yes
Isasi E et al. (2014) [62]	No	No	Yes	Yes	No	No	No	No	Yes	Yes	No	Yes
Rubio-Araiz A et al. (2014) [63]	No	No	Yes	Yes	No	No	No	No	Yes	Yes	No	Yes
Louboutin JP et al. (2010) [67]	No	No	Yes	Yes	No	No	No	Yes	Yes	Yes	Yes	Yes
Estato V et al. (2018) [66]	No	No	Yes	No	No	No	No	No	Yes	No	No	Yes
Bell RD et al. (2010) [69]	Yes	Yes	Yes	No	No	No	Yes	No	Yes	Yes	No	Yes
Li Z et al. (2016) [68]	Yes	Yes	Yes	Yes	Yes	No	Yes	Yes	Yes	Yes	Yes	Yes

This table presents the individual analysis of each paper using the CAMARADES quality assessment format, with slight adjustments. These 12 items assessed the overall quality of the included studies, stating if they were performed and reported (“yes”) or not (“no”).

**Table 4:** Changes in laminin, BBB, and neuroinflammation.

	Author (Year)	Disease Model	Laminin					BBB			BBB Permeability			Neuroinflammation		
			Measurement Time	Technique	Changes		*p* Value	Measurement Time	Technique	Markers	Measurement Time	Technique	Markers	Measurement Time	Technique	Markers
Stroke	McColl BW et al. (2008) [37]	Stroke	4, 8, 24 h	WB, IF	↓ =	No changes in vessel-associated laminin but loss of neuronal laminin at 24 h	--	4, 8, 24 h	WB, IF	Claudin-5 ↓, Occludin =	--	--	--	8–24 h	IF, WB, Zm	MMP-9 ↑
	Ji K, Tsirka SE. (2012) [30]	Stroke	6–12 h	WB, IHC, IF	↑	In WT (α2, β3, and γ1 subunits), but not in COX-2 KO mice	--	--	--	--	6–12 h; 1, 24–48 h	WB, IH	VEGF and CD45 ↑	6 h	WB	TLR9 and CD14 ↑
	Steiner E et al. (2012) [38]	Stroke	12, 24, 48 h	IF	↓	α2 and α4 isotypes decreased at 24 and 48 h; no changes at 12 h	--	24 h	IHC	Claudin-5 =	--	--	--	24, 48 h	IF, WB	β-DG ↓
	Lee JY et al. (2014) [36]	Stroke	7 days	WB, IF	↓	α4 in hippocampus	*p* < 0.05 experimental vs. sham group	7 days	IHC	Occludin ↓	7 days	EVB	Extravasation ↑	7 days	RT-PCR	MMP-2, MMP-9, TNF-α, IL-1β, IL-6, COX-2, iNOS, Gro-α (CXCL-1), MCP-1, MIP-1α, MIP-1β (CCL-4), and MIP-2α (CXCL-2) ↑
	Boroujerdi A et al. (2015) [33]	Chronic hypoxia model	7 days hypoxia, 7 and 14 days post hypoxia	IF	↓	Increased fragmentation of vascular laminin in WT at 7 days hypoxia, and at 7 days and 14 days post hypoxia vs. normoxia	*p* < 0.02 WT 14 days post hypoxia vs. 14 days hypoxia. *p* < 0.005 14 days post hypoxia WT vs. MMP-9 KO	14 days hypoxia. 14 days post hypoxia	IF	Claudin-5 expression in WT ↑ 14 days hypoxia, but ↓ after 14 days post hypoxia	--	--	--	2 d hypoxia and 7–14 d post hypoxia	Zm	MMP-9 levels ↑
	Cisbani G et al. (2018) [34]	Stroke	48 h–7 days	IF	↑	At 7 days in WT, CCR2 KO and CX3CR1 KO	*p* < 0.005 WT and CCR2 KO, *p* < 0.001CX3CR1 KO	--	--	--	48 h–7 days	IF	CD45 48 h–7 days, and 7/4^+^ at 48 h =	7 days	IF	TREM2, TLR2, and CD68 =
	Wang J et al. (2018) [39]	Stroke	3 days	WB, IF	↓	In tMCAO	*p* < 0.05 tMCAO vs. sham	3 days	WB, IF	ZO-1 ↓	6 and 72 h	EVB	Extravasation ↑	3 days	WB	TNF-α, IL1β, and MMP-9 ↑
	Kim DY et al. (2020) [35]	Stroke	16 h	IF, WB	↓	In tMCAO	*p* < 0.001 experimental vs. sham	--	--	--	16 h	EVB	Extravasation ↑	16 h	WB	β-DG ↓
Cerebral Hemorrhage	Suzuki H et al. (2010) [41]	SAH	24 h	WB	↓	In SAH rats	*p* < 0.05 SAH rats vs. sham-operated	24 h	WB	ZO-1 ↓	24 h	EVB	Extravasation ↑ in left and right cerebral hemispheres, cerebellum, and brainstem	24 h	WB	MMP-9 ↑/TIMP-1 ↓, IL-1β ↑
	Zhang XS et al. (2015) [44]	SAH	24 h	WB	↓	In SAH rats	*p* < 0.01 SAH vs. control group	--	--	--	24 h	EVB	Extravasation ↑	24 h	WB, IHC, ELISA	MMP-9, IL-1β, and TNF-α ↑
	Zeng J et al. (2018) [43]	Microhemorrhages	7 days	IF	↓	Laminin	--	7 days	WB	ZO-1 ↓ in the cortex, cerebellum, and the brain stem	7 days	EVB	Extravasation ↑	7 days	IF	Microglia activity ↑
	Gautam, J et al. (2020) [40]	ICH	–	–	↓	In PKO mice	--	2, 5, 14 days	IF	Claudin-5 and ZO-1 =	2, 5, 14 days	EVB, IF	Extravasation and FITC-dextran ↑	5, 14 days	IHC	Microglia activity ↑
	Wang, F et al. (2020) [42]	SAH	72 h	WB	↓	Laminin α2 in SAH	*p* < 0.001 SAH vs. sham group	72 h	WB	Occludin and ZO-1 ↓	72 h	EVB	Extravasation ↑	72 h	WB	VEGFR-2 ↑
Traumatic Brain Injury	Gursoy-Ozdemir Y et al. (2004) [45]	CSD	3–24 h	WB, IHC	↓	Laminin reduced vs. sham group and vs. contralateral side	--	3–24 h	WB, IF	EBA and ZO-1 ↓	3, 6, 12, 24 h	EVB	Extravasation ↑	1, 3, 6, 12, 24, 48 h	RT-PCR, Zm	MMP-9 ↑
	Reyes R et al. (2009) [47]	Thermal injury	7 h	WB	↓	In thermal injury group	*p* < 0.01 experimental vs. control	--	--	--	7 h	IF	FITC-Dextran ↑	7 h	WB, Zm	MMP-9 ↑
	Higashida T et al. (2011) [46]	TBI	24 h	WB	↓	Laminin	*p* < 0.01 TBI vs. control	24 h	WB	ZO-1 and Occludin ↓	24 h	IF	FITC-Dextran ↑	24 h	WB	MMP-9 and HIF-1α ↑
	Tao X et al. (2015) [48]	TBI	6, 24 h	WB	↓	Laminin	*p* < 0.01 experimental vs. control	6, 24 h	WB	Occludin ↓	6, 24 h	EVB	Extravasation ↑	6, 24 h	WB	MMP-9, NF-κB and p65 ↑
	Tao XG et al. (2017) [49]	TBI	6, 24 h	WB	↓	Laminin	*p* < 0.05 experimental vs. control	6, 24 h	WB	Occludin ↓	6, 24 h	EVB	Extravasation ↑	6, 24 h	WB	MMP-9, TNF-α, iNOS and ICAM-1 ↑
Cerebral Ischemia and Alzheimer’s Disease	Hawkes CA et al. (2013) [52]	Stroke (pMCAO)/AD	24 h	IF	↑	In 3 and 12 month old WT and in 3xTg mice with stroke	--	4 h	IHC	CD31 ↓	--	--	--	24 h	IF	Microglia activity ↑
	Amtul Z et al. (2018) [50]	Ischemia/AD	24 h, 28 days	IHC	↑	In the striatum of ET1 and Aβ + ET1	*p* < 0.001 (24 h ET1> Aβ + ET1 rats; 28 days Aβ + ET1 > ET1) vs. control and Aβ rats	--	--	--	1, 7, 28 days	IHC	IgG ↑	24 h	IHC	MMP-9 ↑
	Amtul Z et al. (2019) [51]	Ischemia/AD	4 weeks	IHC	↑	ET1 + Aβ > Aβ > ET1 rats	ET1 + Aβ > Aβ > ET1 rats, *p* = 0.07	4 weeks	IHC	SMI71 ↓	4 weeks	IHC	IgG ↑	4 weeks	IHC	MMP-9 ↑
Alzheimer’s Disease and Aging	Campbell SJ et al. (2007) [53]	Age+Neuroinflammation	6 h	IHC	↓	Age-dependent in striatum	*p* < 0.0001 juvenile vs. young vs. old	6 h	IHC	Claudin-1 ↓	6 h	IHC	IgG ↑	6 h	IHC	IL-1β and TNF-α ↑
	Ryu JK, McLarnon JG. (2008) [54]	AD	7 days	IF	↑	In AD vs. control	*p* < 0.05 AD vs. PBS-injected group	--	--	--	7 days	IF	FITC-albumin ↑	7 days	IF	TNF-α ↑
	Marottoli FM et al. (2017) [55]	AD	24 h	IF	↓	In AD + LPS vs. PBS	*p* < 0.05 E4FAD + LPS vs. PBS	--	--	--	24 h	NaFl	Extravasation ↑	24 h	Cytokine assay	MIP-1, IL-6, IL-5, TNF-α, RANTES, G-CSF, IL-1α, and IL-10 ↑
Multiple Sclerosis	Welser JV et al. (2017) [57]	MS	7, 21, 35 days	IF	↑	At 7 days post EAE immunization vs. controls	At 7 days EAE vs. CFA control (*p* < 0.05). At 21 days EAE vs. CFA control (*p* < 0.01)	21 days	IF	Claudin-5 and ZO-1 ↓	21 days	IF	Fibrinogen leakage ↑	21 days	IF	MHC II^+^ and CD45^+^ ↑
	Girolamo F et al. (2019) [56]	MS	20 days	IF	↑	Laminin ↑ in EAE, and ↓ in NG2 KO and EAE-affected NG2 KO mice	*p* < 0.001 NG2 KO and EAE-affected NG2 KO vs. naïve WT	20 days	IF	Claudin-5 and Occludin ↓	20 days	IF	FITC-dextran ↑	--	MOG immunization	EAE model
Status Epilepticus	Sarkar S, Schmued L. (2010) [60]	SE/Kainic acid	2, 6 days	IHC	↑	In kainic acid	--	2, 6 days	IHC	EBA ↓	--	--	--	2, 6 days	IHC	Microglia activity ↑
	Kim YJ et al. (2014) [58]	vasogenic edema following SE	3–4 days	WB, IHC	↑	In SE	*p* < 0.05 experimental vs. non-SE animals	1, 4 days	IHC	SMI-71 ↓	12 h–4 weeks	WB, IHC	IgG ↑	12 h	IF	Vasogenic edema ↑
	Park H et al. (2019) [59]	vasogenic edema following SE	3 days	WB, IHC	↑	In SE	*p* < 0.05 SE vs. control animals	3 days	IHC	SMI-71 ↓	3 days	WB	IgG ↑	3 days	WB	p38, MAPK, and VEGF ↑
Pharmacological and Toxicological Interventions	Gurney KJ et al. (2006) [61]	LPS	24 h	WB	↓	Laminin α1 levels	*p* < 0.05 WT, *p* < 0.05; KO, *p* < 0.01 vs. saline	24 h	WB	Claudin-5 and Occludin ↓	24 h	14C-sucrose	Extravasation ↑	24 h	Zm, RT-PCR, stereology	MMP-9 ↑
	Urrutia A et al. (2013) [64]	METH	1 h	WB	↓	Laminin	*p* < 0.05 METH vs. saline	--	--	--	1, 3, 24 h	IF	IgG ↑	1 h	WB, Zm	MMP-9 ↑
	Willis CL et al. (2013) [65]	3-chloro-1,2-propanediol	2, 4, 8 days	IF	↑	Irregular laminin profile around the vascular endothelium	--	1, 3 days	WB, IF	Claudin-5 and Occludin ↓	2, 8 days	IF, electron microscopy	Leukocytes and macrophages ↑	1–8 days	IF	CD169 ↑
	Isasi E et al. (2014) [62]	Glutaric acid	30 days	IHC	↓	Laminin	*p* < 0.05 glutaric acid vs. PBS	14, 30 days	WB	Occludin and ZO-1 =	14, 30 days	EVB	Extravasation ↑	--	Postnatal injection	Glutaric acid model
	Rubio-Araiz A et al. (2014) [63]	MDMA	1, 3, 6 h	WB, IHC	↓	Laminin	*p* < 0.01 MDMA vs. saline	--	--	--	3 h	IF	IgG ↑	1–3 h	WB, Zm	MMP-9 and MMP-3 ↑
Pathogen Induced Disease	Louboutin JP et al. (2010) [67]	HIV-1	15 min–6 h	IHC	↓	Laminin	*p* < 0.01 gp120 vs. saline	--	IHC	Claudin-5 ↓	1 h, 24 h	EVB	Extravasation ↑	30 min, 1–24 h	IHC, Zm	MMP-2, MMP-9 ↑
	Estato V et al. (2018) [66]	Toxoplasmosis	40 days	IHC	↓	Laminin	*p* < 0.01 experimental vs. control	--	--	--	40 days	EVB	Extravasation ↑	40 days	IF	Microglia activity ↑
Others	Bell RD et al. (2010) [69]	Pericyte model	6–8 months	WB	↓	In Pdgfrβ^+/−^ and F7 mice	*p* < 0.05 Pdgfrβ^+/−^ vs. F7 mice, and vs. controls	6–8 and 14–16 months	WB	Claudin-5, Occludin, and ZO-1 ↓	1, 6, 8,14,16 months	Fluorometrics	IgG ↑	--	RT-PCR	IL-1β, IL-6, TNF-α, CCL2 and ICAM-1 ↑
	Li Z et al. (2016) [68]	Postoperative cognitive dysfunction	24 h	WB	↑	Laminin	*p* < 0.05 experimental vs. sham	24 h	WB	Occludin and ZO-1 ↓	24 h	ELISA, WB	S100β and IgG ↑	3, 6, 12, 24, 72 h	WB, RT-PCR	MMP-9/TIMP-3, MMP-2/TIMP-3, MMP-9/TIMP-1, MMP-9/TIMP-2. IκBα/IκBα and p-IKKα/β ↑

This table summarizes the findings of all the included articles regarding changes in laminin, BBB or BBB permeability, and neuroinflammation. Arrows pointing up indicate an increase, and arrows pointing down indicate a decrease, while an equal sign indicates no change. In cases where no information was provided, -- is inserted in the table. Abbreviations: Alzheimer’s disease (AD); amyloid beta (Aβ); beta-dystroglycan (β-DG); C–C motif chemokine ligand 2 (CCL2); C–C chemokine receptor type 2 (CCR2); complete Freund’s adjuvant (CFA); cortical spreading depression (CSD); cyclooxygenase-2 (COX2); CX3C chemokine receptor 1 (CX3CR1); experimental autoimmune encephalomyelitis (EAE); endothelial barrier antigen (EBA); endothelin-1 (ET1); enzyme-linked immunosorbent assay (ELISA); Evans blue (EVB); fluorescein isothiocyanate (FITC); granulocyte colony-stimulating factor (G-CSF); growth-regulated oncogene alpha (Gro-α); human immunodeficiency virus (HIV); hypoxia-inducible factor 1 alpha (HIF-1α); intracerebral hemorrhage (ICH); inducible nitric oxide synthase (iNOS); intercellular adhesion molecule 1 (ICAM-1); interleukin (IL); immunofluorescence (IF); immunohistochemistry (IHC); lipopolysaccharide (LPS); macrophage inflammatory protein (MIP); major histocompatibility complex (MHC); matrix metalloproteinase (MMP); methamphetamine (METH); 3,4-methylenedioxymethamphetamine (MDMA); mitogen-activated protein kinase (MAPK); monocyte chemoattractant protein-1 (MCP-1); multiple sclerosis (MS); nuclear factor kappa b (NF-κB); permanent MCAO (pMCAO); phosphate-buffered saline (PBS); platelet-derived growth factor receptor beta (Pdgfrβ); regulated upon activation, normal T cell expressed and presumably secreted (RANTES); real-time polymerase chain reaction (RT-PCR); status epilepticus (SE); subarachnoid hemorrhage (SAH); tissue inhibitor of metalloproteinase (TIMP); Toll-like receptor (TLR); transient MCAO (tMCAO); traumatic brain injury (TBI); triggering receptor expressed on myeloid cells 2 (TREM2); tumor necrosis factor (TNF); vascular endothelial growth factor (VEGF); Western blot (WB); wildtype (WT); zonula occludens 1 (ZO-1); zymography (Zm).

**Table 5:** Changes in cells and in ECM.

	Author (Year)	Disease Model	Laminin			Microglia			Neurovascular Unit Cells			Immune System Markers/Cells			Extracellular Matrix		
			Measurement Time		Changes	Measurement Time	Technique	Marker Changes	Measurement Time	Technique	Marker Changes	Measurement time	Technique	Marker Changes	Measurement Time	Technique	Marker Changes
Stroke	McColl BW et al. (2008) [37]	Stroke	4, 8, 24 h	↓ =	No changes in vessel-associated laminin but loss of neuronal laminin at 24 h	--	--	--	--	--	--	--	--	--	8–24 h	WB, IF	Collagen IV ↓
	Ji K, Tsirka SE. (2012) [30]	Stroke	6–12 h	↑	In WT (α2, β3 and γ1 subunits), but not in COX-2 KO mice	1–48 h	IHC	Iba-1 ↑	--	--	--	1, 24, 48 h	IHC	CD45 ↑	6 h	WB	Fibrinogen and collagen IV ↑
	Steiner E et al. (2012) [38]	Stroke	12, 24, 48 h	↓	α2 and α4 isotypes decreased at 24 and 48 h. No changes at 12 h	--	--	--	12, 24, 48 h	IF, WB	AQP4 ↓ at 24–48 h (= at 12 h) with IF; no changes in WB at 24 h	--	--	--	24–48 h	IF	Agrin ↓
	Lee JY et al. (2014) [36]	Stroke	7 days	↓	α4 in hippocampus	7 days	IF	Iba-1 ↑	7 days	IF, WB	GFAP ↑	--	--	--	--	--	--
	Boroujerdi A et al. (2015) [33]	Chronic hypoxia model	7 days hypoxia, 7 and 14 days post hypoxia	↓	Increased fragmentation of vascular laminin in WT at 7 d hypoxia, and at 7 d and 14 d post hypoxia vs. normoxia	--	--	--	--	--	--	--	--	--	--	--	--
	Cisbani G et al. (2018) [34]	Stroke	48 h–7 days	↑	At 7 d in WT, CCR2 KO and CX3CR1 KO	--	--	--	--	--	--	48 h	FC, IF	CD45 and 7/4+ ↓. Circulating monocytes and neutrophils ↓ in CCR2 KO	--	--	--
	Wang J et al. (2018) [39]	Stroke	3 days	↓	In tMCAO	3 days	IF	Iba-1 ↑	3 days	IF	Pdgfrβ ↓	--	--	--	--	--	--
	Kim DY et al. (2020) [35]	Stroke	16 h	↓	In tMCAO	--	--	--	16 h	IF, WB	GFAP ↑, AQP4 ↑, α-SMA ↓	--	--	--	16 h	IF, WB	β1-integrin, β-DG, and collagen IV/CD31 ↓
Cerebral Hemorrhage	Suzuki H et al. (2010) [41]	SAH	24 h	↓	In SAH rats	--	--	--	--	--	--	--	--	--	--	--	--
	Zhang XS et al. (2015) [44]	SAH	24 h	↓	In SAH rats	24 h	IF	ED-1 ↑	--	--	--	24 h	MPO activity assay	MPO ↑, indicator of neutrophil accumulation	--	--	--
	Zeng J et al. (2018) [43]	Microhemorrhages	7 days	↓	Laminin	7 days	IF	Iba-1 ↑ in LPS	7 days	IF	GFAP ↑ while Pdgfrβ ↓	--	--	--	--	--	--
	Gautam, J et al. (2020) [40]	ICH	–	↓	In PKO mice	5, 14 days	IHC	Iba-1 ↑ in PKO	2, 5, 14 days	IHC	GFAP ↑ while Pdgfrβ ↓	2, 5, 14 days	IHC	Ly6G, CD11b, and CD3^+^ ↑	--	--	--
	Wang, F et al. (2020) [42]	SAH	72 h	↓	Laminin α2 in SAH	--	--	--	72 h	WB	GFAP ↑	--	--	--	--	--	--
Traumatic Brain Injury	Gursoy-Ozdemir Y et al. (2004) [45]	CSD	3–24 h	↓	Laminin reduced vs. sham group and vs. contralateral side	--	--	--	--	--	--	--	--	--	--	--	--
	Reyes R et al. (2009) [47]	Thermal injury	7 h	↓	In thermal injury group	--	--	--	--	--	--	--	--	--	7 h	WB	Collagen IV and fibronectin ↓
	Higashida T et al. (2011) [46]	TBI	24 h	↓	Laminin	--	--	--	24 h	WB	AQP4 ↑	--	--	--	--	--	--
	Tao X et al. (2015) [48]	TBI	6, 24 h	↓	Laminin	--	--	--	--	--	--	6 h, 24 h	Myeloperoxidase activity assay	MPO ↑, indicator of neutrophil accumulation	6 h, 24 h	WB	Collagen IV and integrin β1 ↓
	Tao XG et al. (2017) [49]	TBI	6, 24 h	↓	Laminin	--	--	--	24 h	EM	Astrocyte end-feet swelling	--	--	--	--	--	--
Cerebral Ischemia and Alzheimer’s Disease	Hawkes CA et al. (2013) [52]	Stroke (pMCAO)/AD	24 h	↑	In 3 and 12 month old WT and in 3xTg mice with stroke	24 h	IF	Iba-1 ↑ in pMCAO + 3xTg	24 h	IHC	AQP4 ↓ in 3 month old WT and 3xTg mice; CD31 ↓ 3xTg mice	--	--	--	24 h	IHC	Collagen IV ↑
	Amtul Z et al. (2018) [50]	Ischemia/AD	24 h, 28 days	↑	In the striatum of ET1 and Aβ + ET1	1, 7, 28 days	IHC	OX6 ↑ in ET1 and Aβ + ET1	1, 7, 28 d	IHC	GFAP and AQP4 ↑ in ET1 rats at 7 days and 28 days (compared with Aβ + ET1 rats). GFAP ↓ at 1 day	--	--	--	--	--	--
	Amtul Z et al. (2019) [51]	Ischemia/AD	4 weeks	↑	ET1 + Aβ > Aβ > ET1 rats	4 weeks	IHC	OX6 ↑ in ET1+Aβ > ET1 > Aβ	4 weeks	IHC	βDG, GFAP and AQP4 ↑ in ET1 + Aβ > ET1 > Aβ	--	--	--	--	--	--
Alzheimer’s Disease and Aging	Campbell SJ et al. (2007) [53]	Age + neuroinflammation	6 h	↓	Age-dependent in striatum	--	--	--	--	--	--	6 h	IHC	ED-1 and anti-neutrophil serum ↑	--	--	--
	Ryu JK, McLarnon JG. (2008) [54]	AD	7 days	↑	In AD vs. control	7 days	IF	Iba-1 ↑ in AD	7 days	IF	GFAP ↑ in AD vs. control	--	--	--	--	--	--
	Marottoli FM et al. (2017) [55]	AD	24 h	↓	In AD + LPS vs. to PBS	--	--	--	--	--	--	--	--	--	--	--	--
Multiple Sclerosis	Welser JV et al. (2017) [57]	MS	7, 21, 35 days	↑	At 7 days post EAE immunization vs. controls	21 days	IF	Mac-1 ↑ in β4-EC-KO	--	--	--	21 days	IF	MHC II and CD45 ↑ in β4-EC-KO	--	--	--
	Girolamo F et al. (2019) [56]	MS	20 days	↑	Laminin ↑ in EAE, and ↓ in NG2 KO and EAE-affected NG2 KO mice	--	--	--	20 days	IF	NG2/CD13, Pdgfrβ ↑ in EAE	--	--	--	20 days	IHC	Collagen IV ↓
Status Epilepticus	Sarkar S, Schmued L. (2010) [60]	SE/kainic acid	2, 6 days	↑	In kainic acid	2, 6 days	IHC	CD11b ↑	--	--	--	--	--	--	--	--	--
	Kim YJ et al. (2014) [58]	Vasogenic edema following SE	3–4 days	↑	In SE	--	--	--	3 days–4 weeks	WB, IHC	GFAP ↑	--	--	--	--	--	--
	Park H et al. (2019) [59]	Vasogenic edema following SE	3 days	↑	In SE	--	--	--	3 days	WB, IHC	AQP4 and dystrophin/AQP4 ↓	--	--	--	--	--	--
Pharmacological and Toxicological Interventions	Gurney KJ et al. (2006) [61]	LPS	24 h	↓	Laminin α1 levels	24 h	IF	Iba-1 ↑	24 h	IF	MMP-3 ↑ in Pericytes with desmin in LPS	--	--	--	--	--	--
	Urrutia A et al. (2013) [64]	METH	1 h	↓	Laminin	--	--	--	--	--	--	--	--	--	--	--	--
	Willis CL et al. (2013) [65]	3-chloro-1,2-propanediol	2, 4, 8 days	↑	Irregular laminin profile around the vascular endothelium	--	--	--	2 days	IF	GFAP ↓	1–8 days	IF	CD169 ↑	1–3 days	WB	Fibronectin ↑
	Isasi E et al. (2014) [62]	Glutaric acid	30 days	↓	Laminin	14, 30 days	IHC	Tomato lectin =	30 days	IHC	AQP4 and Pdgfrβ ↓	--	--	--	--	--	--
	Rubio-Araiz A et al. (2014) [63]	MDMA	1, 3, 6 h	↓	Laminin	6 h	IHC	OX-42 ↑	--	--	--	--	--	--	1, 3, 6, 24 h	IF	Collagen IV ↓
Pathogen Induced Disease	Louboutin JP et al. (2010) [67]	HIV-1	15 min–6 h	↓	Laminin	--	--	--	--	--	--	--	--	--	--	--	--
	Estato V et al. (2018) [66]	Toxoplasmosis	40 days	↓	Laminin	40 d	IF	Iba-1 ↑	--	--	--	10, 40, 180 days	IF	Leukocytes ↑ (rolling and adherent)	--	--	--
Others	Bell RD et al. (2010) [69]	Pericyte model	6–8 months	↓	In Pdgfrβ^+/−^ and F7 mice	--	IF, RT-PCR	Iba-1 ↑ in Pdgfrβ^+/−^	--	IF	AQP4 and SNTA1 =	--	--	--	8 months	WB	Collagen IV ↓
	Li Z et al. (2016) [68]	Postoperative cognitive dysfunction	24 h	↑	Laminin	--	--	--	24 h	WB	Pdgfrβ =	--	--	--	--	--	--

This table summarizes the findings of all included articles regarding changes in microglia, other cells part of the neurovascular unit, general immune markers or cells, and extracellular matrix components. Arrows pointing up indicate an increase, and arrows pointing down indicate a decrease, while an equal sign indicates no change. In cases where no information was provided, -- is inserted in the table. Abbreviations: alpha smooth muscle actin (α-SMA); Alzheimer’s disease (AD); amyloid beta (Aβ); aquaporin 4 (AQP4); beta-dystroglycan (β-DG); C–C chemokine receptor type 2 (CCR2); CX3C chemokine receptor 1 (CX3CR1); cortical spreading depression (CSD); cyclooxygenase-2 (COX2); experimental autoimmune encephalomyelitis (EAE); electron microscopy (EM); endothelin-1 (ET1); flow cytometry (FC); glial acidic fibrillary protein (GFAP); human immunodeficiency virus (HIV); immunofluorescence (IF); immunohistochemistry (IHC); intracerebral hemorrhage (ICH); ionized calcium-binding adaptor protein 1 (Iba1); lipopolysaccharide (LPS); major histocompatibility complex (MHC); methamphetamine (METH); 3,4-methylenedioxymethamphetamine (MDMA); multiple sclerosis (MS); myeloperoxidase (MPO); neuron/glia antigen 2 (NG2); platelet-derived growth factor receptor beta (Pdgfrβ); permanent MCAO (pMCAO); real-time polymerase chain reaction (RT-PCR); status epilepticus (SE); subarachnoid hemorrhage (SAH); syntrophin alpha 1 (SNTA1); transient MCAO (tMCAO); traumatic brain injury (TBI); Western blot (WB); wildtype (WT).

## Data Availability

The data that support the findings of this study are available from the corresponding author on request.
